# Target highlights in CASP14: Analysis of models by structure providers

**DOI:** 10.1002/prot.26247

**Published:** 2021-10-10

**Authors:** Leila T. Alexander, Rosalba Lepore, Andriy Kryshtafovych, Athanassios Adamopoulos, Markus Alahuhta, Ann M. Arvin, Yannick J. Bomble, Bettina Böttcher, Cécile Breyton, Valerio Chiarini, Naga babu Chinnam, Wah Chiu, Krzysztof Fidelis, Rhys Grinter, Gagan D. Gupta, Marcus D. Hartmann, Christopher S. Hayes, Tatjana Heidebrecht, Andrea Ilari, Andrzej Joachimiak, Youngchang Kim, Romain Linares, Andrew L. Lovering, Vladimir V. Lunin, Andrei N. Lupas, Cihan Makbul, Karolina Michalska, John Moult, Prasun K. Mukherjee, William (Sam) Nutt, Stefan L. Oliver, Anastassis Perrakis, Lucy Stols, John A. Tainer, Maya Topf, Susan E. Tsutakawa, Mauricio Valdivia‐Delgado, Torsten Schwede

**Affiliations:** ^1^ Biozentrum, University of Basel Basel Switzerland; ^2^ Computational Structural Biology SIB Swiss Institute of Bioinformatics Basel Switzerland; ^3^ Barcelona Supercomputing Center (BSC) Barcelona Spain; ^4^ Genome Center, University of California, Davis Davis California USA; ^5^ Oncode Institute and Division of Biochemistry Netherlands Cancer Institute Amsterdam The Netherlands; ^6^ Bioscience Center, National Renewable Energy Laboratory Golden Colorado USA; ^7^ Department of Pediatrics Stanford University School of Medicine Stanford California USA; ^8^ Microbiology and Immunology Stanford University School of Medicine Stanford California USA; ^9^ Biocenter and Rudolf Virchow Center, Julius‐Maximilians Universität Würzburg Würzburg Germany; ^10^ Univ. Grenoble Alpes, CNRS, CEA, Institute for Structural Biology Grenoble France; ^11^ Program in Structural Biology and Biophysics Institute of Biotechnology, University of Helsinki Helsinki Finland; ^12^ Department of Molecular and Cellular Oncology The University of Texas M.D. Anderson Cancer Center Houston Texas USA; ^13^ Bioengineering Stanford University School of Medicine Stanford California USA; ^14^ Division of Cryo‐EM and Bioimaging SSRL SLAC National Accelerator Laboratory Menlo Park California USA; ^15^ Infection and Immunity Program, Biomedicine Discovery Institute and Department of Microbiology Monash University Clayton Australia; ^16^ Radiation Biology & Health Sciences Division Bhabha Atomic Research Centre Mumbai India; ^17^ Department of Protein Evolution Max Planck Institute for Developmental Biology Tübingen Germany; ^18^ Department of Molecular, Cellular and Developmental Biology University of California, Santa Barbara Santa Barbara California USA; ^19^ Biomolecular Science and Engineering Program University of California, Santa Barbara Santa Barbara California USA; ^20^ Institute of Molecular Biology and Pathology of the National Research Council of Italy (CNR) Rome Italy; ^21^ Center for Structural Genomics of Infectious Diseases, Consortium for Advanced Science and Engineering, University of Chicago Chicago Illinois USA; ^22^ X‐ray Science Division Argonne National Laboratory, Structural Biology Center Argonne Illinois USA; ^23^ Department of Biochemistry and Molecular Biology University of Chicago Chicago Illinois USA; ^24^ School of Biosciences University of Birmingham Birmingham UK; ^25^ Department of Cell Biology and Molecular Genetics Institute for Bioscience and Biotechnology Research, University of Maryland Rockville Maryland USA; ^26^ Nuclear Agriculture & Biotechnology Division Bhabha Atomic Research Centre Mumbai India; ^27^ Department of Cancer Biology University of Texas MD Anderson Cancer Center Houston Texas USA; ^28^ Institute of Structural and Molecular Biology, Birkbeck, University College London London UK; ^29^ Centre for Structural Systems Biology, Leibniz‐Institut für Experimentelle Virologie Hamburg Germany; ^30^ Molecular Biophysics and Integrated Bioimaging Lawrence Berkeley National Laboratory Berkeley California USA

**Keywords:** CASP, community‐wide experiment, cryo‐EM, protein structure prediction, X‐ray crystallography

## Abstract

The biological and functional significance of selected Critical Assessment of Techniques for Protein Structure Prediction 14 (CASP14) targets are described by the authors of the structures. The authors highlight the most relevant features of the target proteins and discuss how well these features were reproduced in the respective submitted predictions. The overall ability to predict three‐dimensional structures of proteins has improved remarkably in CASP14, and many difficult targets were modeled with impressive accuracy. For the first time in the history of CASP, the experimentalists not only highlighted that computational models can accurately reproduce the most critical structural features observed in their targets, but also envisaged that models could serve as a guidance for further studies of biologically‐relevant properties of proteins.

**Abbreviations**
CASP: community wide experiment on the Critical Assessment of Techniques for Protein Structure Prediction
CTD: C‐terminal domain
DHBc: duck hepatitis B core protein
gB: glycoprotein B
HBc: hepatitis B core protein
HBV: hepatitis B virus
hmU: hydoxymethyluracil
HTH: helix‐turn‐helix
JBP1: J‐binding protein 1
JBP2: J‐binding protein 3
J‐DBD: J‐base binding domain
JGT: J‐glucosyltransferase
mAb: monoclonal antibody
PDB: protein data bank
VHCDR: variable heavy chain complementary determining region
VLCDR: variable light chain complementary determining region
VZV: varicella‐zoster virus

## 1 INTRODUCTION

Critical Assessment of Techniques for Protein Structure Prediction (CASP) operation would not be possible without the help of experimental structural biologists, who share their work‐in‐progress with the CASP organization. In the latest round of CASP (CASP14, 2020),^1^ 65 proteins and protein complexes (including an RNA protease H1044 that was split into 10 separate prediction targets) were suggested as modeling targets by 39 structure determination groups from 15 countries. All suggested entries were released for prediction; however, eight of them were canceled, as their structures were not solved in time. Among the solved structures, three were determined by nuclear magnetic resonance (NMR) spectroscopy, seven by (cryo‐EM), and the rest by X‐ray crystallography. The CASP organizers, who are co‐authors of this article, want to thank the experimentalists who contributed to CASP14 (see Table S1), thereby helping to develop more effective protein structure prediction methods.

This article is the sixth in a series of CASP target highlight papers,^2^, ^3^, ^4^, ^5^, ^6^ and the chapters provide accounts of the contributing authors on the accuracy of best models submitted on 12 CASP14 targets (Table 1). All target providers were invited to contribute to the paper, with the exception of five targets structures for which have been solved by using CASP models, described separately in this issue.^7^ The resulting targets presented here include: the neutralizing monoclonal antibody 93k bound to the varicella‐zoster virus fusogen glycoprotein B (H1036 and T1036), the Bacteriophage T5 tail tip complex (H1060 and T1061), polymorphic CDI toxin‐immunity protein complex from *Serratia marcescens* (H1065, T1065s1, and T1065s2), the BIL2 domain from *Tetrahymena thermophila* BUBL1 locus (T1034), BonA from *Acinetobacter baumannii* (T1054), *Caldicellulosiruptor bescii* N4‐cytosine methyltransferase (T1057), The J‐base binding domain of JBP3 (T1068), a cryptic predatory secreted protein Bd0675 from *Bdellovibrio bacteriovorus* (T1074), a small secreted cysteine‐rich protein Tsp1 from *Trichoderma virens* (T1078), histidine zipper coiled coils from *Nitrosococcus oceani, Meiothermus silvanus*, and *Methylobacter tundripaludum* (T1083, T1084, and T1087, respectively), duck hepatitis B core protein (T1099), ASCC1 alkylation response protein from *Alvinella pompejana* (T1101).

**TABLE 1:** The CASP14 target highlights

Target	PDB	Length, aa	Method	Res, Å	Stoichiom	Best model			Runner‐up model		
						GDT	lDDT	QS	GDT	lDDT	QS
H1036	6VN1	856	EM	2.8	A3B3C3			0.77			0.74
T1036s1	6VN1	622	EM	2.8	A1	90.22	0.82		88.12	0.83	
H1060	‐	1106	EM	3.2	A6B3C12D6						
T1061	‐	949	EM	3.2	A3	61.78	0.73		38.85	0.54	
H1065	7M5F	225	X‐Ray	1.59	A1B1			0.69			0.64
T1065s1	7M5F	127	X‐Ray	1.59	A1	95.59	0.90		91.39	0.81	
T1065s2	7M5F	98	X‐Ray	1.59	A1	98.47	0.91		96.17	0.85	
T1034	6Y75, 6TMM	156	X‐Ray	2.3 2.4	A4	93.59	0.85		87.02	0.75	
T1054	6V4V	190	X‐Ray	1.65	A2	92.13	0.87		86.36	0.81	
T1057	7M6B	287	X‐Ray	1.9	A1	94.41	0.90		89.23	0.77	
T1068	‐	211	X‐Ray	1.78	A1	96.09	0.91		61.03	0.56	
T1074	7OC9	202	X‐Ray	1.5	A1	89.77	0.84		60.61	0.58	
T1078	7CWJ	138	X‐Ray	1.25	A2	95.93	0.92		85.66	0.79	
T1083	‐	98	X‐Ray	1.35	A2, coil‐coil	87.77	0.74		87.77	0.74	
T1084	‐	73	X‐Ray	1.93	A2, coil‐coil	92.96	0.84		92.61	0.82	
T1087	‐	93	X‐Ray	1.35	A2, coil‐coil	96.77	0.90		86.02	0.72	
T1099	6YGH	262	EM	3.7	A1	79.07	0.80		56.18	0.62	
T1101	‐	318	X‐Ray	1.4	A1	87.12	0.86		62.29	0.72	

*Note*: Columns indicate target ID, PDB ID, length, experimental method, resolution, stoichiometry, and CASP14 assessment results of the winner and runner‐up model for each target.

The results of the comprehensive numerical evaluation of CASP14 models are available at the Prediction Center web site (http://www.predictioncenter.org). The detailed assessment of the models by the assessors is provided elsewhere in this issue.^9^, ^158^, ^159^, ^160^

## 2 RESULTS

### 2.1 The neutralizing monoclonal antibody 93k bound to the varicella‐zoster virus fusogen glycoprotein B (CASP: H1036 and T1036, PDB: 6VN1). Provided by Stefan L. Oliver, Wah Chiu, and Ann M. Arvin

Members of the *Herpesviridae* are pathogens of humans and animals that cause a wide range of medically and economically important diseases.^8^ The outer lipid membrane of herpesvirus virions is studded with glycoproteins that enable binding to cell membranes and fusion of the virus envelope to initiate entry and establish infection. Herpesvirus orthologs of glycoprotein B (gB) are trimeric proteins that have been classified as type III fusogens due to their structural similarities with vesicular stomatitis virus G protein and baculovirus gp64.^9^, ^10^, ^11^, ^12^, ^13^, ^14^, ^15^ The ectodomain architecture for gB orthologs consists of five structurally distinct domains (DI to V) that fold into a homotrimer with C3 symmetry.

Varicella‐zoster virus (VZV) is an alphaherpesvirus that causes chickenpox (varicella) upon primary infection.^16^ VZV establishes latency in sensory ganglion neurons and subsequent reactivation manifests as shingles (zoster). In addition to virion entry fusion, characteristic polykaryocyte formation caused by cell–cell fusion within tissues in vivo is essential for VZV pathogenesis. This process can be modeled in vitro via syncytia formation of VZV infected cells in culture.^17^, ^18^ Critically, there are adverse health effects directly linked to cell fusion between differentiated host cells; fusion between ganglion neurons and satellites has been associated with postherpetic neuralgia, and strokes have been linked to vascular endothelial cell fusion.^19^, ^20^, ^21^

**FIGURE 1:**
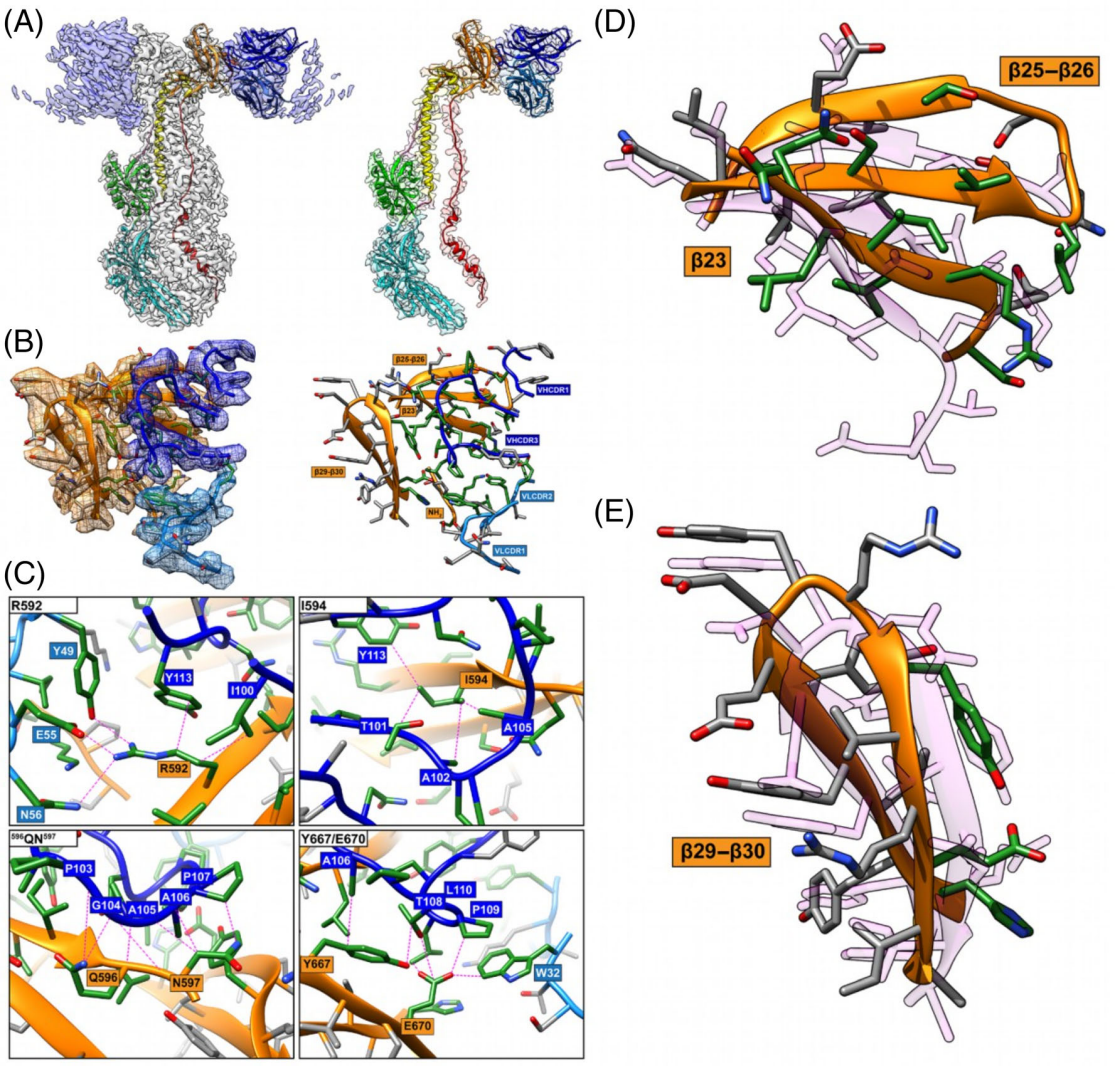
Comparison of the RaptorX model to critical amino acid side chains identified at the gB‐93k interface. Images (A) to (C) were reproduced from Oliver et al., 2020 under the Creative Commons Attribution 4.0 International license. (A) Near‐atomic resolution (2.8 Å) cryo‐EM structure of human neutralizing mAb 93k Fab fragments bound to native VZV gB. The left panel shows the cryo‐EM map gB trimer (gray) and the 93k Fab fragments (blue) with the underlying model for one gB protomer and a 93k Fab fragment. Domains are colored accordingly: (DI; cyan), (DII; green), (DIII; yellow), (DIV; orange), (DV; red), linker regions (hot pink), 93k VH (blue) and 93k VL (light blue). The left panel shows a segmentation of the cryo‐EM map for one VH and VL chain of a 93k Fab fragment bound to a protomer of VZV gB. The structures of VZV gB and 93k VH and VL are represented as ribbons. (B), Extracted density (left panel) for the gB‐93k interface. The densities of gB DIV (orange), 93k VH chain (blue) and 93k VL chain (light blue) are highlighted. A ribbon diagram and side chains of the amino acids at the extracted densities are shown with those highlighted in green representing the interactions formed at the gB‐93k interface. The right panel duplicates the left panel but without the extracted cryo‐EM map densities. The β23, β25–26, β29–30, and the NH_2_ terminus of gB are highlighted with orange boxes, and the VHCDR1, VHCDR3, VLCDR1, and VLCDR2 are highlighted by blue boxes; VH—dark blue, VL—light blue. (C), Molecular interactions between gB and the VH and VL chains of mAb 93k. The four panels show the interactions between gB residues R592, I594, ^596^QN^597^, and Y667/E670D with mAb 93k. Dotted lines (magenta) represent molecular interactions calculated using Find Contacts (Chimera). (D) and (E) Comparison of RaptorX model T1036s1TS487_1‐D1 to the cryo‐EM structure of β23, β25‐26, and β29‐30, where mAb 93k binds to VZV gB. The RaptorX model is shown in pink

The functional domains of herpesvirus gB orthologs have been characterized using monoclonal antibodies (mAbs) that neutralize viral infection via binding to gB before membrane fusion.^11^, ^22^, ^23^, ^24^, ^25^, ^26^, ^27^, ^28^, ^29^, ^30^, ^31^ Although the molecular interactions for some of these antibodies with gB residues have been defined previously, it was unknown whether these gB residues were involved in fusion function or virus infection.^11^, ^28^ A newly derived human mAb, 93k, neutralized VZV by binding to gB and membrane fusion inhibition.^32^ To elucidate gB domain function and their role in VZV infection, a 2.8 Å resolution cryo‐EM structure of native, full‐length VZV gB in complex with mAb 93k Fab fragments was determined.^32^ This near‐atomic resolution structure revealed residues within gB DIV that were then shown to be essential for membrane fusion by evaluating DIV mutants in a virus free assay. Mutagenesis of the VZV genome demonstrated their significance for gB fusion functions necessary to produce infectious extracellular VZV virions and for cell fusion to form syncytia. These findings are highly relevant for developing novel therapies that inhibit infection by disrupting gB DIV‐dependent molecular mechanisms of cell entry or cell fusion by members of the *Herpesviridae*. The interactions between the variable heavy chain complementarity‐determining region 3 (VHCDR3) loops of 93k and gB DIV were the most important features to be modeled by CASP14 participants. These interactions became evident from the 2.8 Å cryo‐EM map (Figure 1A–C),^32^ providing near‐atomic level details of the mAb 93k footprint on VZV gB. Sidechains of I100, A102, A105, and Y113 from VHCDR3 formed a hydrophobic network with gB residues R592 and I594 of β23, and V617 and L619 of β25 (Figure 1C; see supplemental movie 3 in Oliver et al.^32^). The aromatic ring of VHCDR3 Y113 formed a cation‐π interaction with gB R592 that was inserted into a negatively charged pocket within the 93k antigen binding site. In addition, the OH group of VHCDR3 Y113 and the side chain of N111 the carbonyl oxygen formed H‐bonds with and backbone nitrogen of gB I593 and L595, respectively (Figure 1C). At the boundary of gB β23 and 93k interface the carbonyl oxygens of VHCDR3 P103 and G104 H‐bonded with the side chain of the gB Q596 and the backbone nitrogen of N597, respectively, while the backbone nitrogen of VHCDR3 A106 H‐bonded with the gB L595 carbonyl oxygen (Figure 1C). The gB‐93k interface made a sharp turn where hydrophobic and Van der Waals contacts dominated the 93k interaction with gB β28–30. The H‐bond between VHCDR3 T108 OG1 and gB E670 OE1 was surrounded by hydrophobic interactions between residues P107, P109, and L110 of VHCDR3, and W32 of the variable light chain CDR1 (VLCDR1) and gB β28–30 residues F655, H658, V660 and Y667 (Figure 1C). This complex network of hydrophobic and hydrophilic interactions at the gB‐93k interface of postfusion gB identified the strongest interactions between gB β23 and β30, and the 93k VHCDR3. Importantly, because mAb 93k has neutralizing activity through fusion inhibition,^32^ residues within gB DIV β23 and β30 were implicated in a functional role for membrane fusion. Indeed, two or more alanine substitution of residues within β23 and β30 reduced or abolished fusion and limited the capacity of VZV to infect cells, indicating that these residues act together to ensure that the gB structure supports its fusion function.

For CASP14, VZV gB from PDB 6VN1 was released as only a monomeric target for server predictors (T1036s1), and separately as a whole target (gB‐93k) for multimeric modeling (H1036). The existence of multiple structures for herpesvirus gB orthologs meant that the server‐only target in T1036s1 was easily modeled. The top five automatic servers generated models with GDT‐TS scores between 86 and 90. The best model was generated by the RAPTOR‐X server (T1036s1TS487_1‐D1) and with a GDT‐TS score of 90 (CA RMSD 1.56 Å over the entire gB protomer). However, the two regions containing gB β23, β25–β26, and β29–β30, primarily involved in mAb 93k binding, were poorly modeled, with RMSD of 5.05 Å (182 atom pairs ^589^SDTRIILQN^597–613^LISIVSLNGSGTVEGQ^628^) and 3.06 Å (145 atom pairs ^658^HYVYYEDYRYVREIA^672^), respectively (Figure 1D,E). Although the overall topology of β23, β25–β26, and β29‐β30 was modeled with some level of accuracy, it did not match the cryo‐EM structure. This was unsurprising given the structural variability of DIV for herpesvirus gB orthologs compared to VZV.^32^, ^33^

None of the multimeric modelers in H1036 was able to correctly place the Fab fragments of 93k bound to gB. The top two predictions for the complete complex, three gB protomers and three 93k Fabs, according to the F1, QS, and Jaccard scores, were H1036T403_2 (F1 = 71.7; QS = 0.668; Jaccard = 0.74) and H1036TS191_5 (F1 = 71.2; QS = 0.769; Jaccard = 0.72). Although the similarity scores for the complex were high (IDDT of 0.763 and 0.756; TM of 0.705 and 0.702, respectively) these were attributed to the scaling effect of gB as it dominates the complex due to its larger size (931aa) compared to the mAb 93k Fabs (VH—128aa; VL—107aa). In addition, gB was modeled well with most of the input to the whole complex fold scores arising from gB. Heterotrimers (H1036v0) were also evaluated. However, the Jaccard coefficient and F1 score were poor, <0.45, meaning that only 45% of the interface and inter‐chain contacts are reproduced. The difficulty in accurately modeling mAb 93k likely arises due to the requirement of gB for the correct folding of mAb 93k VHCDR1, VHCDR3, VLCDR1, and VLCDR2. For example, VHCDR3 (^100^ITAPGAAPTPLNFYG^114^), which is critical for mAb 93k binding to gB, was inadequately modeled for H1036T403_2 and H1036TS191_5 with RMSD of 4.63 Å and 3.52 Å, respectively. Thus, the intermolecular interactions at the VZV gB and mAb 93k interface could only be determined experimentally with current state‐of‐the‐art cryo‐EM methodologies.

### 2.2 Bacteriophage T5 tail tip complex (CASP: H1060 and T1061, PDB: N/A). Provided by Romain Linares and Cécile Breyton

Bacteriophages are the most abundant biological organisms on Earth. As bacterial viruses, they have an utmost impact on the regulation, diversity, evolution, and pathogeny of all bacterial populations. The large majority of bacteriophages are composed of a capsid, which protects the viral double stranded DNA, and a tail, which serves to recognize the host, perforate its cell wall, and safely deliver the viral genome into the bacterial cytoplasm. The mechanism of cell wall perforation is relatively well documented for *Myoviridae*, phages with a long contractile tail, and related contractile injection bacterial systems.^34^ Much less is known in the case of *Siphoviridae*, phages that bear a long, flexible tail, which represent 60% of all phages.

**FIGURE 2:**
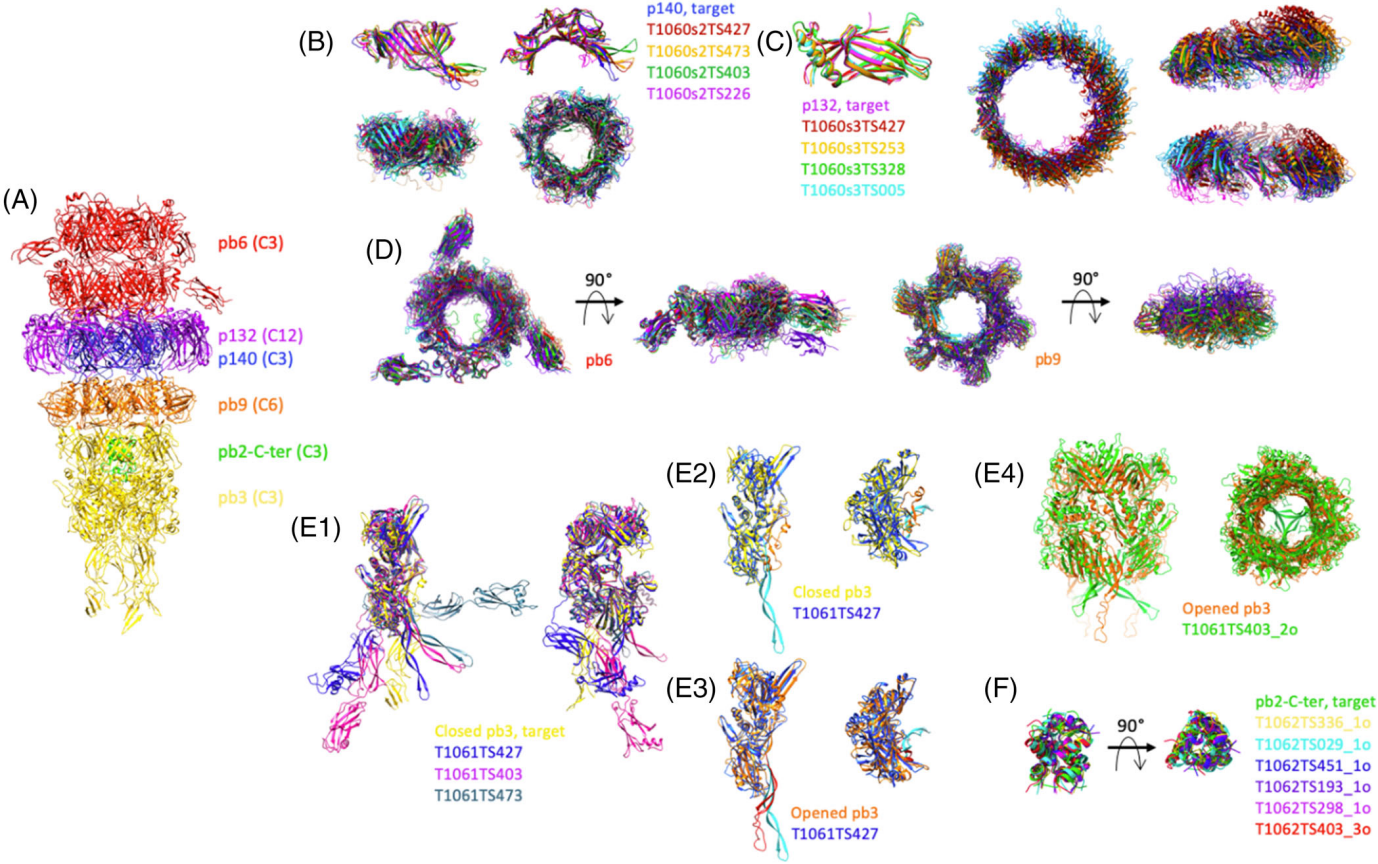
Comparison of the predicted structures of individual proteins and sub‐complexes thereof, of the tip of phage T5. (A) Experimental structure of phage T5 tail tip. The two pb6 rings are colored red, that of p140: blue, the p132 dodecamer: purple, the pb9 hexamer: orange, the pb3 trimer: yellow and the pb2‐C‐terminus: green. The symmetry of each protein is indicated in brackets. (B) Monomer (top) and trimer (bottom) predictions for p140. Side (left) and top (right) views. (C) Monomer (left) and dodecamer (right) predictions for p132. The dodecamer is shown in a top view and two side views, rotated by 90°. (D) Ring prediction for pb6 trimer (left) and pb9 hexamer (right). Top and side views. (E1) Monomer predictions for pb3, two side views, rotated by 90°. (E2 and E3) T1061TS427 prediction was superimposed either on the closed pb3 (E2) or on the open pb3 (E3). The 35‐residue plug is colored orange in the closed pb3 and red in the open one, and cyan in the T1061TS427 prediction. Side (left) and top (right) views. (E4) Trimer prediction of pb3. The open form of pb3 (orange) is shown rather than the closed one. In (E2–E4), the fibronectin domains have been removed for clarity. (F) trimeric predictions for pb2. In each panel, predicted structures are superimposed on the target, shown in the same color as in Panel (A). Superimpositions are done with the Chimera tool MatchMaker. In the case of a multimer, the structures are aligned to one monomer

We are interested in understanding the molecular mechanism of cell wall perforation in the case of the sipho‐coliphage T5. At the distal end of its tail, T5 bears a tail tip complex that is formed by the collar, to which are attached three L‐shaped fibers and a conical structure that is prolonged by a straight fiber at which end is located T5 Receptor Binding Protein pb5.^35^ The irreversible binding of pb5 to FhuA, an *Escherichia coli* outer membrane transporter, commits T5 to infection: it induces cell wall perforation and opening of the capsid. Using cryo‐electron microscopy, we determined the structure of T5 tail tip, before and after interaction with its receptor FhuA^36^: we could solve the structure of two rings of the Tail Tube Protein pb6, prolonged by a ring of p140 surrounded by a dodecamer of p132 that forms the collar, a hexameric ring of pb9, a trimeric ring of pb3, which closes the tube, and a trimer of the C‐terminus of the Tape Measure Protein, pb2 (Figure 2). Although the structures of pb9 and pb6 were already available,^37^, ^38^ structures of p140, p132, pb3, and pb2 were unknown.^35^ The structure of the whole tail tip before interaction with the receptor has been submitted to CASP14. The pb6–p140–p132–pb9 complex has been proposed to the competition, as well as the individual rings and individual proteins.

Although not having any sequence homology with pb6, p140 shares the same fold^36^ and both form a trimeric ring. This was well predicted, with the best GDT‐TS = 83 for the monomer, and a QS‐score of 0.442 for the trimeric ring. The inner‐ring diameter was correctly reproduced in the best quality model only, while it was predicted to be smaller in all other models (Figure 2B). The structure of p132 monomer, which belongs to the immunoglobulin superfamily, was very well predicted (best GDT‐TS = 95). The dodecameric ring was also well predicted by five groups (QS‐scores from 0.442 to 0.228). The predicted models contained more or less altered subunit interfaces, resulting in slightly smaller rings and/or modified subunit orientation within the ring (Figure 2C). For both p140 and p132, AlphaFold2 is far ahead of the others (by 18 and 24 points on the GDT‐TS parameter). Pb6 and pb9 rings were also well predicted, with best QS global scores of 0.650 for pb9 hexameric ring and 0.379 for pb6 trimeric ring (Figure 2D). At least the six top groups predicted the correct inner diameter of the tube, even though the orientation of the protein within the ring is not always optimal, due to modified subunit interactions.

An important protein of this assembly is pb3, which closes the tube. This protein is predicted to share structural similarity with the baseplate hub proteins of *Myoviridae* and related contractile injection bacterial systems.^35^ It is, however, a larger protein, with in addition two fibronectin domains in C‐terminus predicted from the sequence.^35^ Indeed, the protein is composed of the four canonical “hub domains” (HDs) of phage T4‐pg27,^39^ with a large insertion in the second one to allow the closure of the tube, and two C‐terminal fibronectin domains (Figure 2E1). Only three groups predicted the structure of the four HDs correctly, with GDT‐TS values of 62 for the top group, AlphaFold2, and 39 for the two others. While the relative position of the fibronectin domains with the rest of the protein was not predicted correctly by either of the groups, this was not surprising given the 30 residue‐long linker, and the absence of interactions with neighboring subunits (Figure 2E1). Very interestingly, these predicted structures do not represent pb3 in its closed conformation, in which part of the insertion in HDII is folded back along the inner wall of the tube to provide a plug to close the tube (orange in Figure 2E2). This plug sequence (45 residues) is rather stretched out downwards as a long beta hairpin in the predicted structures (cyan in Figure 2E2). This is very close to the structure of pb3 after interaction of the tail with its receptor, which induces the opening of the tube (Figure 2E3), which thus seems to represent a more stable conformation of the protein (unpublished results). When the pb3 trimer is considered, only one group predicted it with satisfaction and here again in the open conformation (Figure 2E4; QS‐score with the closed pb3 trimer was 0.252). Others, even with similar QS scores, did not predict the correct monomer structure. The trimeric pb2‐C‐terminal helical bundle was very well predicted by six groups, with QS‐scores ranging from 0.678 to 0.607 (Figure 2F).

**FIGURE 3:**
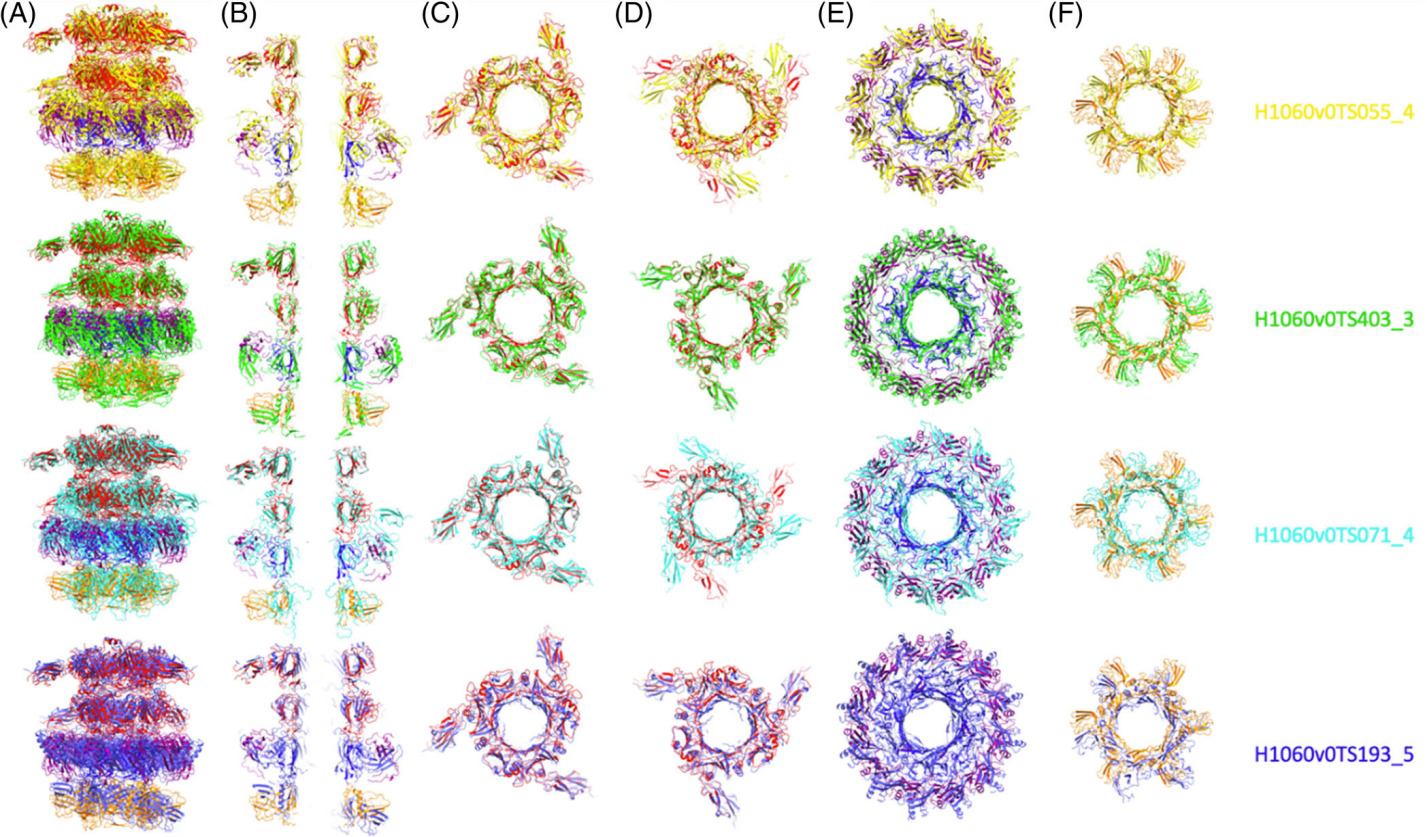
Four best CASP predictions of phage T5 tail tip complex (H1060v0, pb6‐pb6‐p140‐p132‐pb9) aligned on the experimental structure, in which the different proteins are colored as in Figure 2. (A) Side view of the complex and (B) central slice of the complex. Top views of the different rings of the complex from its top to its bottom with (C) a first, and (D), second trimers of pb6, (E) the p140 trimer (inner ring) and p132 dodecamer (outer ring) and (F) the pb9 hexamer

With regards to the pb6–p140–p132–pb9 complex, four groups predicted reasonably the general tube assembly (QS‐score of 0.266–0.196), with the correct inner‐tube diameter and inter‐ring distances. Inter‐ring interactions were however not optimal, as none predicted the correct register of the different rings (Figure 3).

In conclusion, each target (whether it was monomers, rings, or full complex) was reasonably well predicted by at least one CASP14 competitor, and very often by several ones. The best structure predictions for p132, p140, and pb3 monomers were highly accurate, as well as for the pb2 trimer. In the case of ring assemblies, although some predictions were reasonably close to the targets, it was surprising to observe noticeable variations regarding ring diameter/orientation, and structure predictions of the monomers within the rings were not as good as the ones of the monomers alone. As for the full complex, four groups made acceptable predictions of the global architecture (global QS‐score of 0.266–0.196, see Figure 3), including ring diameter and orientation, inter‐ring distance and monomer relative positions, but did not predict the correct register. Another interesting point is that pb3 monomer was predicted in a conformation which is closer to its open conformation, indicating a probable more stable state. We also witnessed that AlphaFold2 systematically outperformed other competitors on monomeric targets.

### 2.3 Structure of polymorphic CDI toxin‐immunity protein complex from *S. marcescens* (CASP: H1065, T1065s1, T1065s2, PDB: 7M5F). Provided by Karolina Michalska, Youngchang Kim, William (Sam) Nutt, Lucy Stols, Christopher S. Hayes, and Andrzej Joachimiak

Many Gram‐negative bacteria deploy “contact‐dependent growth inhibition” or “CDI” systems to inhibit the growth of competitors in environmental niches.^40^, ^41^ CDI has been characterized extensively in *E. coli* and other Gram‐negative bacteria, and these systems are particularly common in pathogenic species.^41^, ^42^, ^43^, ^44^, ^45^ CDI systems have also been shown to mediate cooperative behaviors—such as biofilm formation, persistence, and virulence—between isogenic sibling cells.^46^, ^47^, ^48^, ^49^ CDI loci encode toxic CdiA effector proteins, which are used to inhibit the growth of neighboring bacteria; and CdiI immunity proteins that protect CdiA producing cells from auto‐intoxication. CdiA is exported to the cell surface, where it forms an elongated filament that extends to interact with receptors on susceptible target bacteria. CdiA undergoes a series of complex conformational changes that result in delivery of its C‐terminal toxin region (CdiA‐CT) into the target cell.^50^ The sequence of the CdiA‐CT region is highly variable between bacteria, and this polymorphism corresponds to many distinct toxin activities. The Center for Structural Genomics of Infectious Diseases has worked with the biology community to determine the structures and functions of several CDI toxin‐immunity protein complexes. Although most CDI toxins characterized to date have nuclease activities, there is a considerable range of substrate specificities. Some CDI toxins are nonspecific DNases^51^ or RNases,^52^ whereas several others are tRNases that specifically cleave 16S rRNA^53^ or individual tRNA isoacceptors.^54^, ^55^, ^56^

**FIGURE 4:**
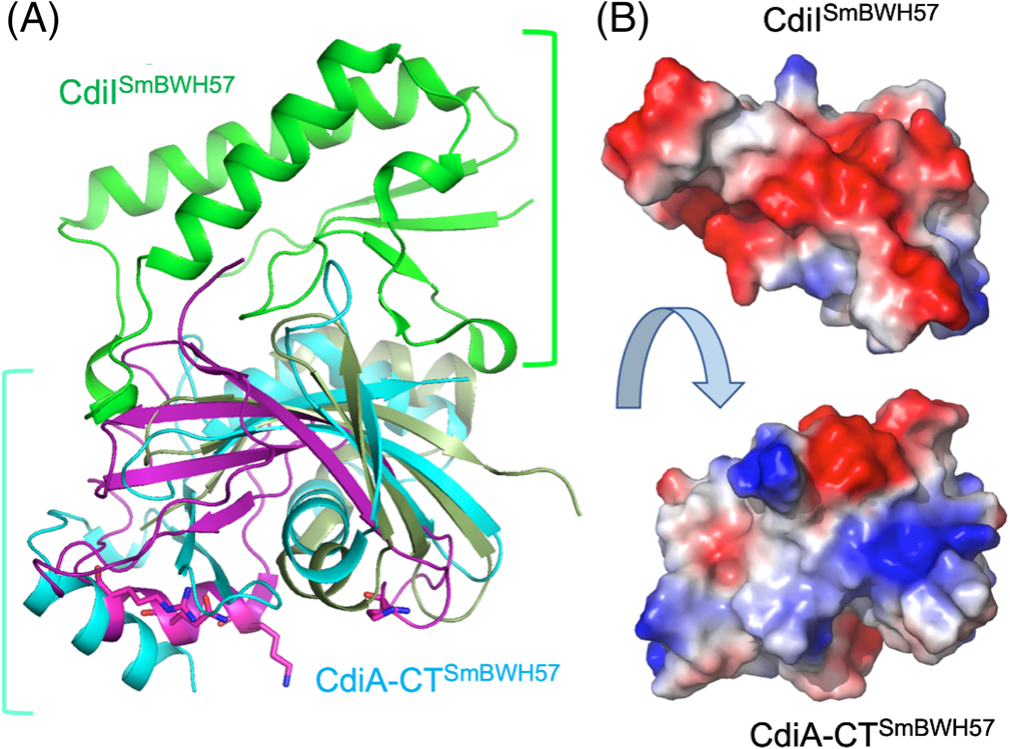
The crystal structure of CdiA‐CT^SmBWH57^•CdiI^SmBWH57^ complex. (A) The toxin component (cyan) is compared with the RNase domain of histidine‐free ribonuclease colicin E5 (magenta) and BrnT toxin from *Brucella abortus* (dark green). The C‐terminal domain of CdiA‐CT covers approximately half of the RNase protein. The key residues in the active site of colicin E5 are shown as sticks. There are no equivalent residues in CdiA‐CT^SmBWH57^. The CdiI^SmBWH57^ (green) does not have any close structural homologs in PDB. The CdiI^SmBWH57^ interacts tightly with toxin using different surface on opposite site of colicin E5 active site. (B) Interacting surfaces of CdiA‐CT^SmBWH57^and CdiI^SmBWH57^ are highly complementary in terms of shape and charge potential

We recently determined the high‐resolution crystal structure of a novel CDI toxin‐immunity protein complex from the nosocomial pathogen *S. marcescens* BWH57 (Figure 4). The CdiA‐CT^BWH57^ region is ~280 residues in length and is composed to two domains. The N‐terminal domain of CdiA‐CT^BWH57^ shares 69% sequence identity with the corresponding domain in CdiA‐CT_o11_^EC869^ from *Escherichia coli* EC869 (PDB: 4G6U).^51^ This N‐terminal domain is required for toxin translocation into the cytosol of target bacteria,^57^ but this region is not resolved in the CdiA‐CT•CdiI^BWH57^ complex structure as it was cleaved off by in situ proteolysis prior to crystallization. The C‐terminal domain has homologs in over 900 predicted antibacterial proteins found in *Serratia*, *Yersinia*, *Pantoea*, *Listeria*, and other genera. The CdiI^BWH57^ immunity protein is 98 residues and is broadly distributed with nearly 1000 family members in γ‐proteobacteria, β‐proteobacteria, and cyanobacteria. This complex is an excellent target for the CASP competition, because the component proteins have no sequence homologs in the PDB, and the activity of the CdiA‐CT^BWH57^ toxin domain and its interactions with CdiI^BWH57^ are not easily predicted. The CdiA‐CT^BWH57^ toxin domain adopts the Barnase/EndoU/Colicin/RelE (BECR) RNase superfamily fold (Figure 4),^56^, ^58^ though it has no detectable sequence similarity to known BECR enzymes and is not annotated as such. The DALI server identified several structural homologs for the toxin including: MqsR from *E. coli* K‐12 (PDB: 3HI2),^59^ BrnT from *Brucella abortus* (PDB: 3U97),^60^ isoacceptor‐specific CDI tRNase toxins from *Klebsiella pneumoniae* 342 and *E. coli* NC101 (PDB: 6CP9, 5I4Q),^55^, ^56^ and the C‐terminal nuclease domains of colicin E5 (PDB: 2DJH)^61^ and colicin D (PDB: 1V74).^62^ In contrast, DALI identified only very distant structural homologs of CdiA‐CT^BWH57^, suggesting that it may represent a new protein fold.

The CdiA‐CT^BWH57^ nuclease domain includes three α‐helices and one 3_10_ helix, four antiparallel β‐strands arranged in a small concave β‐sheet and two β‐strands that form a hairpin. The β‐sheet and β‐hairpin wrap around α4, which serves as a core of this fold. Helix α3 has a significant kink and helix α1 interacts with the β‐hairpin. CdiI^BWH57^ has a simple α/β fold with two α‐helices, three 3_10_ helices and four mixed β‐strands arranged in a small β‐sheet. The toxin's interaction surface is largely electropositive and complemented by a negatively charged patch on the immunity protein (Figure 4B). CdiI^BWH57^ binds to the nuclease domain using the large loop linking β1 to β2 and three 3_10_ helices (Figure 4). These secondary structure elements interact with the exposed β‐sheet residues, two loop regions, helix α3, and the C‐terminus of the toxin domain. Several CdiI^BWH57^ residues that interact with the toxin, including K5, D9, Y10, W16, D25, and the C‐terminal Y98, are highly conserved across the protein family. Similarly, toxin residues H47, E51, H52, R89, N117, and R119 that interact with the immunity protein are also highly conserved. A subset of these latter residues (H47, E51, H52, R89) are good candidates to form the nuclease active site, suggesting that CdiI^BWH57^ binding to the toxin blocks access to its RNA substrates.

**FIGURE 5:**
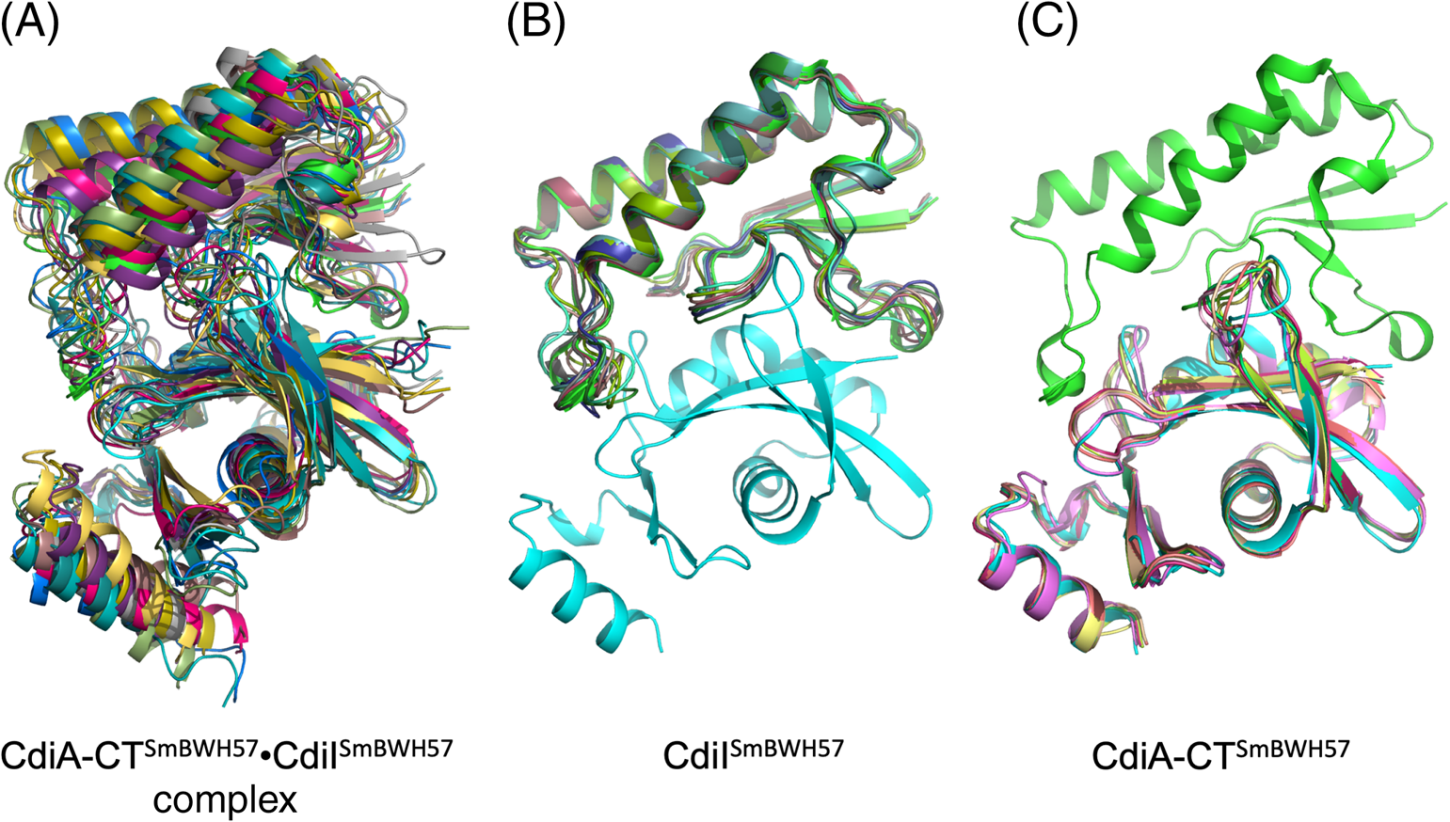
Comparisons of the crystal structure of CdiA‐CT^SmBWH57^•CdiI^SmBWH57^ complex with 10 best models predicted in CASP14. (A) Predictions of the CdiA‐CT^Sm BWH57^•CdiI^SmBWH57^ complex. (B) Predictions of CdiI^SmBWH57^ and (C) predictions of CdiA‐CT^SmBWH57^

For the CASP14 competition, CdiA‐CT^BWH57^ and CdiI^BWH57^ were first modeled as individual monomers, and the top 10 predictive models, as ranked by GDT‐TS score, were evaluated. Figure 5 shows these top 10 models for CdiA‐CT^BWH57^, CdiI^BWH57^, and the complex superimposed with the crystal structure. Predictions of CdiI^BWH57^ were of high quality, with the top 10 models showing GDT‐TS scores of higher than 90.56. The best model (T1065S2TS427) had an outstanding GDT‐TS score of 98.47 over the entire length of the protein, correctly predicting the length and orientation of the α and 3_10_ helices, the location of the β‐sheet and the conformation of the loop regions (Figure 5B). The only large deviation from the crystal structure was at the C‐terminus of CdiI^BWH57^. Models for CdiA‐CT^BWH57^ were also very good with the top 10 models showing GDT‐TS scores of 90.13 or higher over 119 of 120 residues. The top model (T1065S1TS427) showed 95.59 GDT‐TS score and correctly predicted the α and 3_10_ helices, including the kink in α3. The shape of the β‐sheet was also modeled well, including the conformation of the loop regions. The only large deviation from the crystal structure was in the hairpin region and the loop connecting two β‐strands (Figure 5C).

Predictions of the CdiA‐CT•CdiI^BWH57^ complex were clearly more challenging. The top 10 models (Figure 5A) had GDT‐TS scores between 64.47 and 74.88 over 225 residues. Interestingly, one model of the complex placed the CdiA‐CT^BWH57^ nuclease domain in a wrong orientation. The best model (H1065TS192) correctly predicted the individual proteins and the interaction surface. Interestingly, its GDT‐TS score of 74.88 was much lower than that of the best model for the isolated CdiI^BWH57^ protein (T1065S2TS427, GDT‐TS score 95.59). Therefore, if the best predictions for the individual components were used, models for the binary complex would likely improve.

### 2.4 BIL2: *Holo* structure from *apo* sequence (CASP: T1034, PDB: 6Y75, 6TMM). Provided by Valerio Chiarini and Andrea Ilari

Inteins are invasive protein domains translated together with N‐terminal and C‐terminal host protein fragments, called N and C exteins, respectively. Upon translation, they are able to catalyze a reaction known as protein splicing, which allows the intein to escape from the homing protein while joining the two exteins without leaving any trace of the intein insertion. This mechanism does not compromise the host protein functionality, and inteins are maintained and passed down as harmless genomic elements.

The canonical protein splicing reaction takes place in four steps.^63^ Initially, the intein's first residue (C1) forms a (thio‐)ester by replacing the backbone amino group in the peptide bond connecting the intein with the N‐extein (N—S acyl shift). In the second step, the (thio‐)ester is transferred on to the first residue of the C‐extein, forming a branched intermediate (transesterification). In the third step, the intermediate is resolved by the cyclization of the last intein residue (N), inducing a C‐terminal cleavage that frees the intein from the joined exteins. In the final step, the thioester connecting the exteins is then rearranged to peptide bond (S—N acyl shift).

An intein BIL2 is a part of the polyubiquitin locus of *T. thermophila*. It is flanked by two independent ubiquitin‐like domains (ubl4/ubl5).^64^, ^65^ BIL2 catalyzes protein splicing with a peculiar mechanism that leads to the formation of an isopeptide bond (K[εNH2]‐C‐ter). Previously, we demonstrated that BIL2 operates as a “single‐ubiquitin‐dispensing‐platform,” allowing the conjugation of ubl4 to different substrates such as ubl5 and Ras GTPase.^66^ Since the splicing reaction is ATP‐independent, the presence of the intein allows the host to avoid employing energy‐consuming cascades of enzymes usually deputed to ubiquitin conjugation.

**FIGURE 6:**
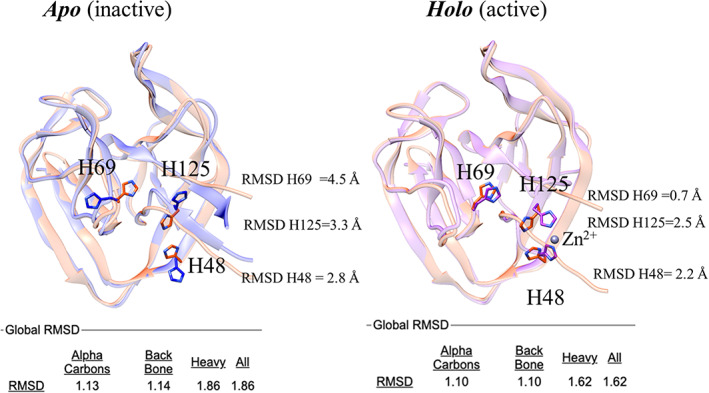
Structure superposition of the 3D model from AlphaFold2 (salmon) with the BIL2 experimentally determined structures (cornflower blue and magenta). The catalytic residue H69, as well as the Zn coordinating residues H125 and H148 residues, are highlighted in sticks. Labels indicate per‐residue RMSD values over backbone and side chains (calculated with CHIMERA). Global RMSD values (calculated with Superpose) over alpha carbons, backbone, and side chains atoms are reported in the bottom part of the figure

In order to elucidate the molecular mechanism of BUBL protein splicing, we solved the high‐resolution crystal structures of BIL2 in both *apo* and zinc‐bound forms. The analysis of the structures revealed that zinc induces a conformational change of H69, which has been suggested to function as a key catalytic residue,^67^ to a position that stabilizes the N/S acyl shift intermediate and thereby activates protein splicing. Intein's catalytic residues are located at the N‐ and C‐termini where the cleavage occurs. Hence, both the correct folding and the orientation of the residue H69, which putatively acts as a proton exchanger^68^, ^69^ during the first step of protein splicing (the *N*‐acyl shift), are the necessary conditions for the intein's function. In deposited PDB intein structures, the side chain of H69 points toward the intein C1 residue. Interestingly, for our BIL2 experimental structures this was not the case. In the *apo* state, the H69 side chain pointed away from C1 in a unique conformation (Figure 6). On the contrary, in the *holo* state, where BIL2 binds a Zn atom, H69 adopts the typical catalytic conformation.

Splicing assays in presence or absence of ligands demonstrated the inhibitory effect of Zn binding on inteins in several studies.^70^, ^71^, ^72^ While for such inhibition the mechanism remains elusive despite the availability of a few *holo* structures, in the case of BIL2, the activation of H69 is remarkably explicit upon the binding of Zn. Because we were able to identify at least two different Zn‐binding sites across the ubiquitin‐like domains, we speculate that the binding induces a conformational change that allows the nuclophilic lysine of ubl5 to be correctly placed at the N‐terminal splicing junction, leading to isopeptide formation.

In CASP14, BIL2 was correctly modeled by most predictors, with model generated by AlphaFold2 being the most accurate (GTD‐TS of 93.59), followed by MULTICOM and BAKER groups (GTD‐TS of 87.02 and 86.70, respectively). Despite the high quality of the predictions, the differences between the models and our two experimental structures highlighted the dependencies that algorithms still have on the PDB as a training set.

We believe this dependency might be the reason why none of the top 10 CASP algorithms predicted the novel “inactive” orientation of the residue H69 (closest conformation from RaptorX, followed by Yang_FM). At the same time, the side chains of both Zn‐binding residues (H48 and H125) were modeled correctly, as if they were indeed binding the Zn ligand. While the predictors did not fully replicate the features of the *apo* structure, the *holo* conformation was modeled with exceptional accuracy, supporting the evidence that the binding of Zn is in fact structurally and functionally coupled to the catalytic orientation of H69.

In conclusion, the lack of information about Zn binding did not prevent the predictors from inferring the structural conformation coupled to the active state of the intein. Although BIL2 is a relatively small domain with a known fold, these results highlight an unprecedented ability to predict biologically relevant features with atomic‐level details.

### 2.5 Structure of BonA from *A. baumannii* (CASP: T1054, PDB: 6V4V). Provided by Rhys Grinter

BonA is an outer‐membrane lipoprotein from the opportunistic pathogen *A. baumannii* that is important for maintaining the structure and function of the outer membrane.^73^ In *A. baumannii*, the loss of BonA causes the loss of cell motility and a change in the structure of the outer membrane.^73^ BonA homologs in other bacterial species (designated YraP or DolP) form part of the cell envelope stress regulon (e.g., SigmaE regulon in *E. coli*).^74^ These BonA homologs are important for the integrity of the outer membrane and the virulence of bacterial pathogens (e.g., *Neisseria gonorrhoeae*, *Salmonella enterica*).^75^, ^76^, ^77^ BonA and its homologs localize to the divisome, the large protein complex that mediates cell division in bacteria.^75^, ^78^ As part of the divisome, DolP, the BonA homolog from *E. coli*, regulates the activity of cell wall remodeling enzymes during cell division.^79^ The mechanism by which BonA and its homologs mediate their function remains unknown.

BonA is 235 amino acids in length and is composed of two Bacterial OsmY and Nodulation (BON) domains BON1 and BON2. Each of them is approximately 75 amino acids long and folds into a conserved α/β sandwich.^75^, ^80^ In addition to its dual‐BON domains, BonA possesses a proline‐rich 45 amino acid C‐terminal extension, which is absent from most of its homologs. BonA is tethered to the membrane by an N‐terminal cysteine‐linked acyl chain, which is connected to the first BON domain by a 27 amino acid linker. BonA forms a decamer, composed of a pentamer of dimers, with the 27 amino acid N‐terminal linker playing an important but undefined role in decamer formation.^73^

At the time of solving the structure of BonA and of CASP14, there were no structurally characterized homologs available. The structure of DolP, a distantly related BonA homolog from *E. coli*, was subsequently solved by NMR.^75^ BON domains are not thought to function as enzymes, as they contain no known conserved catalytic motifs. However, protein structural information can provide insight into cryptic actives sites, not easily discernible from analysis of amino acid sequence alone. Additionally, the initial purification and analysis of BonA showed that it forms a decamer.^73^ Other BON domain‐containing proteins had not previously been shown to oligomerize, so it was unclear how the decamer of BonA formed. By determining the structure of BonA, I aimed to identify possible functional motifs and understand its oligomerization to establish the underlying mechanism for its role in the bacterial cell envelope.

**FIGURE 7:**
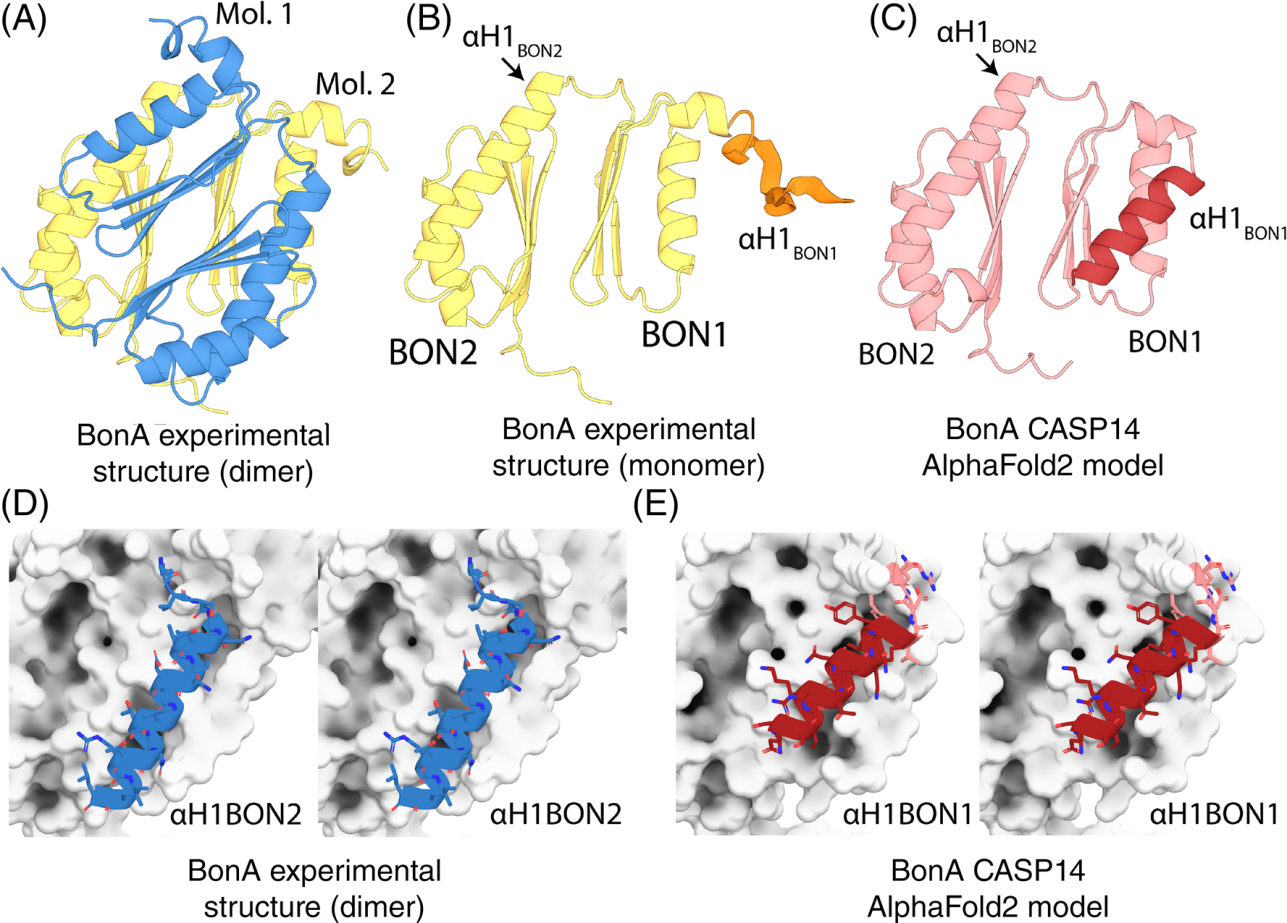
Comparison of the BonA experimental structure and CASP14 model. (A) The X‐ray crystal structure of BonA‐27N, showing the symmetrical dimer observed in the crystal structure. (B) One molecule of the BonA‐27N dimer shown in Panel A, with α‐helix 1 of BON1 (αH1 of BON1), which is disordered in the crystal structure, modeled as an unstructured polypeptide in orange. (C) The CASP14 AlphaFold2 model of BonA, with αH1 BON1 that adopts a canonical BON‐domain fold highlighted in dark red. (D) A cross‐eye stereo view of the interaction between αH1 of BON2 of one molecule of the BonA dimer (shown as a blue cartoon), with its dimer partner (shown as a white surface) in the experimentally determined structure. (E) A cross‐eye stereo view of the interaction between αH1 of BON1 (shown as a red cartoon) and the remainder of the monomeric BonA AlphaFold2 model, showing the αH1 of BON1 adopts an analogous conformation to that of αH1 of BON2 in the experimental structure

After unsuccessful attempts to crystallize full‐length BonA, minus its N‐terminal lipid anchor, several truncated BonA variants were generated for crystallization. This resulted in the determination of the structure of BonA minus its N‐terminal 27 amino acid linker (BonA‐27 N). This structure was solved by experimental phasing, due to a lack of suitable homologous structures. In contrast to full‐length BonA, in solution, BonA‐27N exists as a monomer.^73^ However, in the crystal structure, BonA‐27N formed a dimer (Figure 7A), that has an extensive buried surface area of 3236 Å^2^ according to PISA.^81^ In the BonA‐27N structure, the C‐terminal BON domain (BON2) adopts the canonical α/β‐sandwich fold, consisting of two α‐helices and three β‐sheets. However, in the N‐terminal BON domain (BON1), α‐helix 1 is displaced from the α/β‐sandwich, by α‐helix 1 of BON2 from the opposing dimeric molecule, which forms a hydrophobic interaction that facilitates dimer formation (Figure 7B). I hypothesized that this dimer was a constituent of the BonA decamer and performed additional structural analysis of full‐length BonA using small‐angle X‐ray scattering and negative stain electron microscopy, revealing that the decamer was pentameric, consisting of five BonA dimers.^73^

The sequence corresponding to BonA‐27N was submitted as a target for CASP14 (CASP ID: T1054). A number of CASP14 participants produced models that were very accurate when compared to the experimentally determined structure of BonA‐27N. For regions of the sequence resolved in the crystal structure, six groups obtained GDT‐TS of >80. While the model produced by AlphaFold2 was unambiguously the most accurate (GDT‐TS = 92.1), both this model and a number of the other top scorers served as successful models for molecular replacement of the BonA‐27N experimental data (including those from the FEIG‐R3, ProQ2, and LamoureuxLab groups). As the AlphaFold2 model was the most accurate, it was utilized for detailed comparison with the experimental structure of BonA‐27N (Figure 7C). The region of the BonA model corresponding to BON2 very closely reproduced the experimental data, with an RMSD of 0.4 Å. The 45 amino acid C‐terminal extension of BonA, which was disordered in the experimental structure, was also accurately modeled as an unstructured polypeptide. The model also reproduced the orientation of BON1 relative to BON2 with high accuracy, giving an overall model‐to‐experimental RMSD of 0.97 Å (Figure 7C).

A major difference between the model and experimental data was the orientation of α‐helix 1 of BON1, which rather than being displaced from BON1 as in the experimental structure, adopted a canonical BON domain conformation (Figure 7C). This position of α‐helix 1 of BON1 in the model precludes the formation of the dimer observed in the crystal structure and is analogous to BON1 of DolP, which exists as a monomer when purified.^75^ Experimental evidence indicates that BonA is stable as a monomer both when purified and in the bacterial cell.^73^ To exist in this state, the hydrophobic surface protected by α‐helix 1 of BON2 in the dimer would need to be shielded from the solvent (Figure 7D). α‐helix 1 of BON1 in the CASP14 models adopts analogous conformation to α‐helix 1 of BON2 (Figure 7E), corresponding to the monomeric form of BonA. Thus, while disagreeing slightly with the experimental structure, the predicted model of BonA most likely represents a physiologically relevant conformation of the protein.

In summary, CASP14 produced highly accurate models of BonA, a challenging target for which only one very distantly related structural template was available. Further, in addition to reproducing the experimental structure, these models may provide additional insight into the dynamics of this protein.

### 2.6 Structure of *C. bescii* N4‐cytosine methyltransferase (CASP: T1057, PDB: 7M6B). Provided by Markus Alahuhta, Vladimir V. Lunin, and Yannick J. Bomble

*C. bescii* α‐class N4‐cytosine methyltransferase (M.CbeI) is a thermostable DNA restriction enzyme that is required for transformation of *E. coli* DNA to *C. bescii*.^82^ The ability to genetically engineer this thermophilic and naturally cellulolytic organism is important for consolidated bioprocessing (CBP) of biomass to biofuels and biochemicals. M.CbeI is structurally somewhat similar (RMSD 2.64 Å and secondary structure similarity of 43% by PDBeFold [https://www.ebi.ac.uk/msd-srv/ssm/]) to *E. coli* DNA adenine methyltransferase (PDB: 4RTR) but shows no sequence similarity with any characterized N4‐cytosine methyltransferase. We determined the structure of M.CbeI to characterize the possible unique structural features of this enzyme. Unfortunately, we were unable to crystallize it with DNA which resulted in an open conformation that likely does not represent the catalytically competent conformation of the enzyme.

**FIGURE 8:**
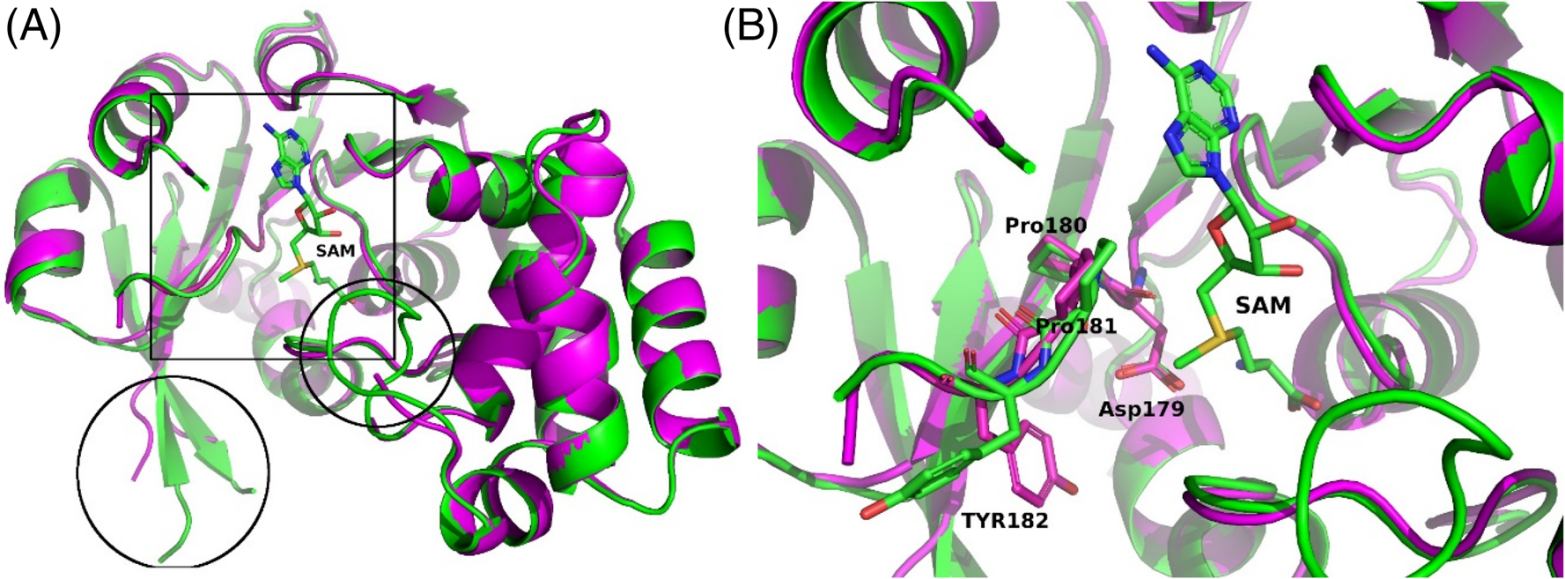
Model T1057TS427 superimposed with M.CbeI. (A) Overall view highlighting main differences (box: active site region and circles: regions with disorder/differences). (B) Close up view of the active site showing the conserved DPPY motif and SAM in stick representation. M.CbeI is shown as a green ribbon and green carbon atoms when cartoon representation is used. Model T1057TS427 is shown in magenta. Blue nitrogen atoms, red oxygens and yellow sulfur atoms are used for both in stick representation. Figures were made using PyMol^83^, ^84^, ^85^ and GIMP 2.10.18 (http://gimp.org/)

The highest ranked model T1057TS427 reproduced the overall structure of M.CbeI very well (GDT‐TS 94.41; Figure 8A). Some of the loop regions showed increased variability as expected but closer inspection showed the active site to be very similar to the X‐ray structure (IDDT 0.90, all atom RMSD 0.512 Å). The conserved DPPY motif (Asp179, Pro180, Pro181, and Tyr182) of this methyl transferase was well modeled with almost no main chain shift. This model would lead to correct positioning of active site residues. Most importantly the Asp179 side chain was correctly modeled (Figure 8B), while the Tyr182 side chain was predicted to be in a different conformation compared to the experimentally determined structure This is understandable due to the flexibility of this loop hinge region, but it should be noted that this flipped conformation is similar to the *E. coli* DNA adenine methyltransferase structure (PDB code 4RTR) where the S‐adenosyl methionine (SAM) cofactor is bound in a different conformation compared to M.CbeI. The authors of this model likely mainly relied on structure comparison and did not minimize their model with SAM as part of their model.

The second ranked model T1057TS335 also had a very similar overall structure (GDT‐TS 89.23, all atom RMSD 0.759 Å) as the highest ranked model T1057TS427. The active site area was highly similar to the M.CbeI X‐ray structure. Interestingly, this model correctly reproduced the position of the Tyr182 side chain in addition to the other three DPPY motif residues. Overall, the top two models correctly reproduced the tertiary fold and the secondary structure of M.CbeI. They correctly formed the active site cleft with only minor differences. Both models would allow correct assignment of active site catalytic residues and environment. When looking at the rest of the models, the first 65 models had GDT‐TS scores above 75 and the 65th ranked model T1057TS342 at GDT‐TS 75.2 still had an all atom RMSD of 1.395 Å with active site residues approximately in correct positions but with increased main chain shifts compared to the two highest ranked models.

### 2.7 The J‐base binding domain of JBP3 (CASP: T1068, PDB:N/A). Provided by Athanassios Adamopoulos, Tatjana Heidebrecht, and Anastassis Perrakis

The modified DNA nucleotide β‐d‐glucopyranosyloxymethyluracil (base J) replaces 1% of the thymine (T) nucleotides in kinetoplastid protozoa. 99% of base J is found in telomeric repeats; the remaining 1% has a functional role in transcription termination.^86^ Base J is specifically recognized by JBP1, a protein central to the epigenetic replication and biosynthesis of base J. JBP1 recognizes base J by a short (~150 residues) J‐base DNA‐binding domain (J‐DBD), which adopts a helix‐turn‐helix (HTH) fold that we previously described.^87^ JBP1 binds base J DNA, and preferentially hydroxylates a T 13 base pairs downstream (but not upstream) on the complementary DNA strand.^88^ T hydroxylation results in hydroxymethyluracil (hmU), which is the substrate for the transfer of a glucose moiety to hmU by J‐glucosyltransferase (JGT), resulting in base J. Recently, it has been shown that a new protein, now named JBP3 owing to the existence of a domain homologous to J‐DBD, binds to JGT and other transcription complexes, contributing to transcription regulation in protozoa.^89^, ^90^

JBP3 J‐DBD binds both J‐DNA and normal DNA with similar low μΜ affinity, and shows limited 2–4 fold preference toward J‐DNA. This is in sharp contrast to JBP1 J‐DBD that binds J‐DNA with low nM affinity in vitro, and has a remarkable discrimination against normal DNA, which it binds with μΜ affinity. The low sequence identity between the JBP1 and JBP3 J‐DBD domains (16.5%) was enough to establish the homology between them, but not sufficient to understand their difference in J‐DNA specificity from sequence conservation alone. Importantly, Asp525, the JBP1 residue that we have previously shown to be crucial for discriminating J‐DNA against normal DNA, is conserved, as well as Lys522A and Arg532A (but not Lys518 or K524), which are all important for general DNA binding.

We therefore decided to determine the structure of the J‐DBD domain of JBP3, to understand what are the structural determinants that confer the limited affinity and specificity toward J‐DNA. We were surprised to find out that we were unable to determine the structure of the JBP3 J‐DBD by molecular replacement. We determined the structure using massive combination of small fragments and density modification as implemented in Archimboldo–Lite.^91^ The main difference between the JBP1 and JBP3 J‐DBD domain structures is the placement of the N‐terminal region and C‐terminal helix (α5) of the helical bouquet fold that we have previously described. The N‐terminal region of JBP3 J‐DBD (~35 residues) was adopting an entirely different orientation compared to the core HTH fold compared to the JBP1 J‐DBD, while the C‐terminal helix was placed in an angle of ~90 degrees compared to its positioning in the JBP1 J‐DBD. The HTH recognition helix(α4) harboring the crucial Asp525 residue, connects to the C‐terminal helix of the JBP1 J‐DBD through a loop containing the key Arg‐532 residue involved in DNA‐recognition, thus its different placement is of particular interest. To this point, we still do not fully understand why JBP3 has limited discrimination between J‐DNA and normal DNA, or the functional importance of this adaptation.

While a few methods in CASP14 predicted well the relative orientation of the two longest helices of the fold (α1, α2) and some predicted fairly well (albeit not accurately) also the relative orientation of the support (α3) and recognition helices (α4), they all failed to model accurately the N‐terminal region and the orientation of the C‐terminal α5 helix. The highest GDT‐TS score for these methods was 61.03, with a total of 30 methods produced models with scores higher than 55.0. Rather remarkably, the score of the AlphaFold2 model was 96.09. AlpaFold2 correctly predicted the different placement of both terminal regions in relationship to the rest of the fold, but also modeled with remarkable accuracy the relative placement of the α1, α2 pair of helices in relation to the α3, α4 pair. Importantly, this could be independent evidence that the relative placement of the N‐terminal region and helix α5 were not an artifact of crystal packing, but a real feature of the structure of JBP3 J‐DBD. Finally, the AlphaFold2 model, but not other models we examined, is sufficiently accurate to easily phase the crystallographic data using a standard version of PHASER.^92^

### 2.8 A cryptic predatory secreted protein, Bd0675, from *B. bacteriovorus* (CASP: T1074, PDB: 7OC9). Provided by Mauricio Valdivia‐Delgado and Andrew L. Lovering

*B. bacteriovorus* are ubiquitous predatory Gram‐negative δ‐proteobacteria which predate on other Gram‐negative bacteria.^93^ To succeed as a micro‐predator, *B. bacteriovorus* has developed a lifecycle consisting of location of the prey and initiation of the attack phase, attachment, and entry of prey, invasion of periplasm, bdelloplast formation, filamentous growth, exhaustion of prey cell resources, septation, and bdelloplast lysis and release of progeny.^93^, ^94^ The transcriptomic analysis of *B. bacteriovorus* strain HD100 has shown the upregulation of ~240 genes during predation (the predatosome), and the roles of most are cryptic and require further examination.^95^

We obtained the 1.50 Å structure of Bd0675 (target T1074), a 14 kDa secreted cryptic predatosome protein with no discernible domain annotation. Proteins similar to Bd0675 are found in different *B. bacteriovorus* strains and in other predators such as *Halobacteriovorax* spp.,^96^ but no information regarding their function is available.

The determined structure, covering 133 of the 134 residues of the mature protein, forms a β‐roll‐like distorted architecture containing two α‐helices and nine β‐strands (Figure 10A). The overall β‐roll fold part of the structure is formed by two β‐sheets, one comprised by β‐strands 1–4, which is connected to a second sheet, comprising β‐strands 5–9, via a disulphide bond formed between residues C31 and C132, which appears to adopt two alternative conformations. Additionally, a disulphide bond C90–C118 links the 19 residues loop between β‐strands 6 and 7 with β‐strand 8, suggesting that correct positioning of this loop is relevant for Bd0675 function. All cysteine residues are conserved in predatory homologs.

**FIGURE 9:**
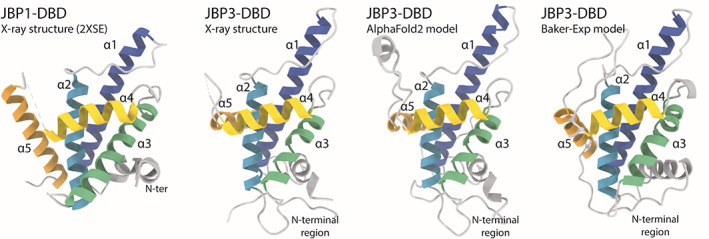
Structures of J‐DNA binding domains. From left to right: the structure of JBP1‐DBD is the closest homolog to the CASP14 target crystallographic structure of JBP3‐DBD that is shown next to it; the AlphaFold2 model has all independent evidence in essentially identical orientation as the experimental structure; the second best model (from the Baker group) shows a different orientation for the α5 helix and the N‐terminal region

**FIGURE 10:**
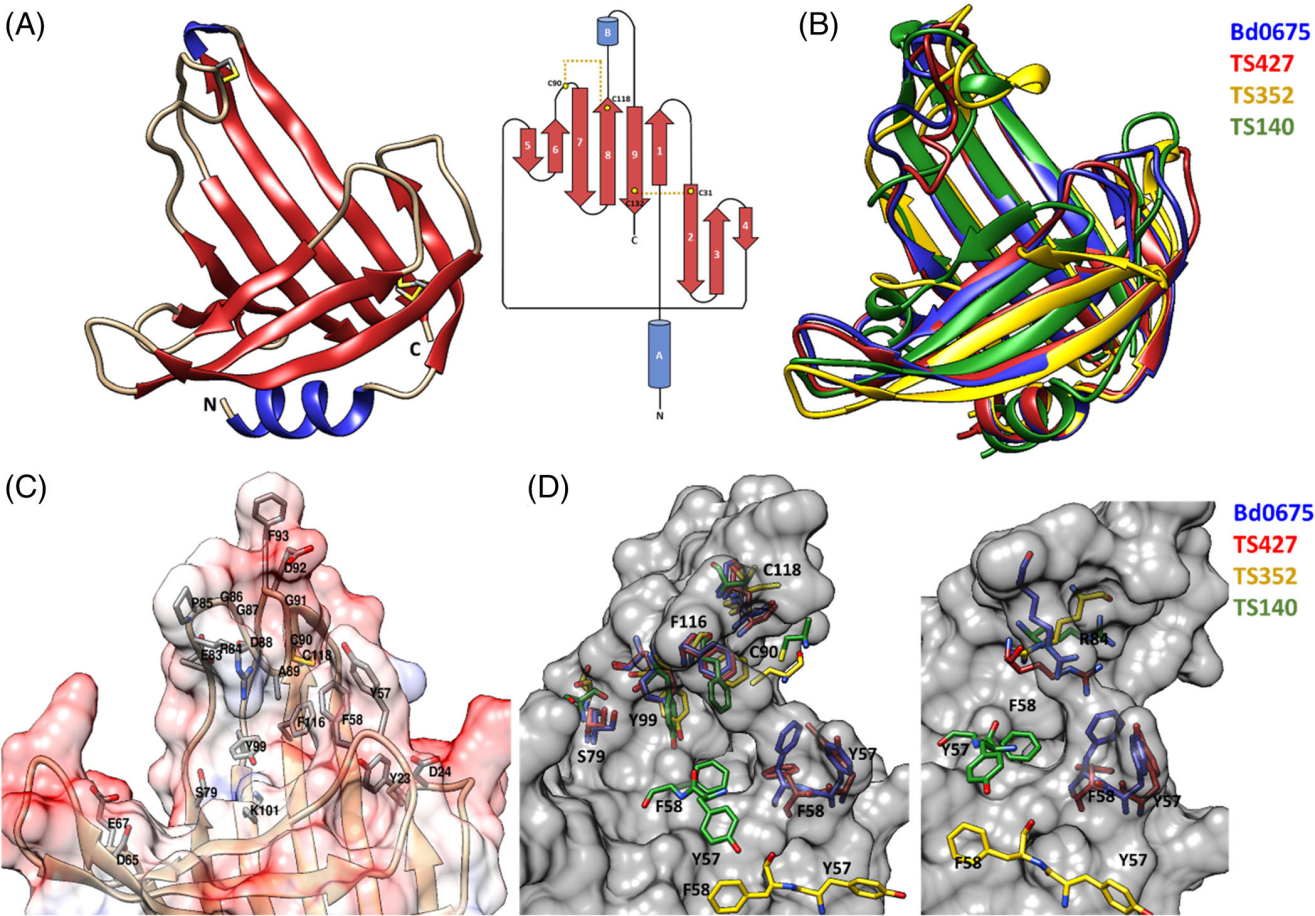
(A) Crystal structure and topological diagram of Bd0675 showing a β‐roll like architecture stabilized by two disulphide bonds (in yellow). (B) Superposition of Bd0675 crystal structure (blue) and the three best models: TS427 (red), TS352 (yellow), and TS140 (green). (C) Surface potential representation of the putative ligand binding cleft. The proposed ligand binding groove of Bd0675 is formed mainly by acidic and hydrophobic residues. (D) Comparison of the proposed ligand binding site of the Bd0675 crystal structure and the three best models

An electrostatic surface potential shows that Bd0675 possesses a hand‐like shape with a potential binding cleft, which is mainly negatively charged, situated in the middle of the protein formed by the connecting loops between β‐strand C‐termini ends, where the outermost loop is fixed in position by the C90–C118 disulphide bond, forming part of a (R/D)PGGXφCGXΩX_5_Y motif, where X is any amino acid, φ is a hydrophobic residue and Ω is an aromatic residue. Residues Y23 and D24 (loop β1–2), Y57 and F58 (loop β3–4), D65, E67 (loop β4–5), and residues 83–93 (β6–7), form lobes limiting the width of the proposed ligand binding site of Bd0675 (Figure 9C). The limiting lobes are mostly composed of negatively charged and aromatic side chains in addition to residues S79, Y99, and K101 which are located toward the center of the cleft. Besides L12 (which forms part of the hydrophobic core) and G14 (located in the loop connecting α‐helix A with β‐strand 1), and the four cysteines, the only invariable residues of Bd0675 are Y57, G86, G87, and G91. Y57 localizes toward one of the delimiting loops of putative groove, forming a pocket with Y23, Y53, F58, and L128 (Figure 10). The conservation of this pocket could assist in Bd0675 function, which remains to be characterized.

Remarkably, models predict the Bd0675 main fold features observed in the crystal structure to a high standard despite the lack of homologous proteins in the PDB or a large number of related sequences (Figure 10B), particularly TS427 (GDT‐TS = 91.10), TS352 (GDT‐TS = 60.61) and TS140 (GDT‐TS = 56.44). Two out of the three best scoring models, TS427 and TS352, predict the correct positioning of the disulphide bond between C31 and C132, however, only model TS427 correctly positions the C90–C118 disulphide. Residues S79, Y99, and F116, oriented toward the putative ligand binding groove, are depicted in different conformations for the predicted models with exception of TS427, superimposing accurately with the crystal structure of Bd0675. Even so, TS427 does not successfully model the experimentally determined orientation of residues Y57 and F58, which are invariant in related predators and therefore suggested to be key in Bd0675 biological function (Figure 9D). Furthermore, the interaction between residues F58 and R84 is proposed to contribute to the positioning of the disulphide stabilized loop and thus, delimiting the ligand binding cleft. In this regard, none of the models correctly predict the conformation of the F58‐R84 pair, contributing to the imprecisions of the disulphide loop of Bd0675 models.

### 2.9 Structure of a small, secreted cysteine‐rich protein Tsp1 from *T. virens* (CASP: T1078, PDBID: 7CWJ). Provided by Gagan D. Gupta and Prasun K. Mukherjee

Plant innate immune response can broadly be divided into two groups; molecular pattern triggered immunity (PTI) and effector triggered immunity (ETI).^97^ In PTI, the pathogen associated molecular patterns are recognized by the plant receptors and is the first line of defense for the plants. If the microbes are able to cross this first barrier, then the effector molecules secreted by the microbes induce the immune response (ETI). *Trichoderma* species are important biocontrol agents used in agriculture. These fungi colonize the roots, promote plant growth, and provide protection to host plants from other phytopathogens.^98^, ^99^ However, little is known at molecular level, how the immunity is triggered in plants. Small secreted cysteine rich proteins (SSCPs) play important role in fungi‐host interaction and are known to act as microbial signaling molecules (elicitor/effector).^100^, ^101^ Many effector/elicitor proteins are secreted by *Trichoderma* that enables it to develop a symbiotic relationship with plant and to induce defense.^102^ Tsp1, an SSCP of hitherto unknown function, from *T. virens* was identified in the secretome analysis post 96‐h interaction of this fungus with maize (host) roots.^103^, ^104^ Tsp1 was the only SSCP that was upregulated upon colonization while as many as 13 other SSCPs were downregulated at this time point. The protein is very well conserved in Ascomycota division of fungi, but none of its homologs have been characterized yet. We have determined the crystal structure of Tsp1 to elucidate its function. A high‐resolution structure has been obtained (1.6 Å) using Se‐SAD methods. Tsp1 adopts β‐barrel fold and forms dimer in crystalline state, which was also observed in solution form using gel‐filtration chromatography.^103^ No enzymatic activity has been observed for the protein. The dimerization seems to be biologically relevant and might be required for binding to a host cell receptor. All four cysteines in Tsp1 sequence form intra‐chain di‐sulfide bonds, providing additional stability to the protein in extra cellular space.^103^

**FIGURE 11:**
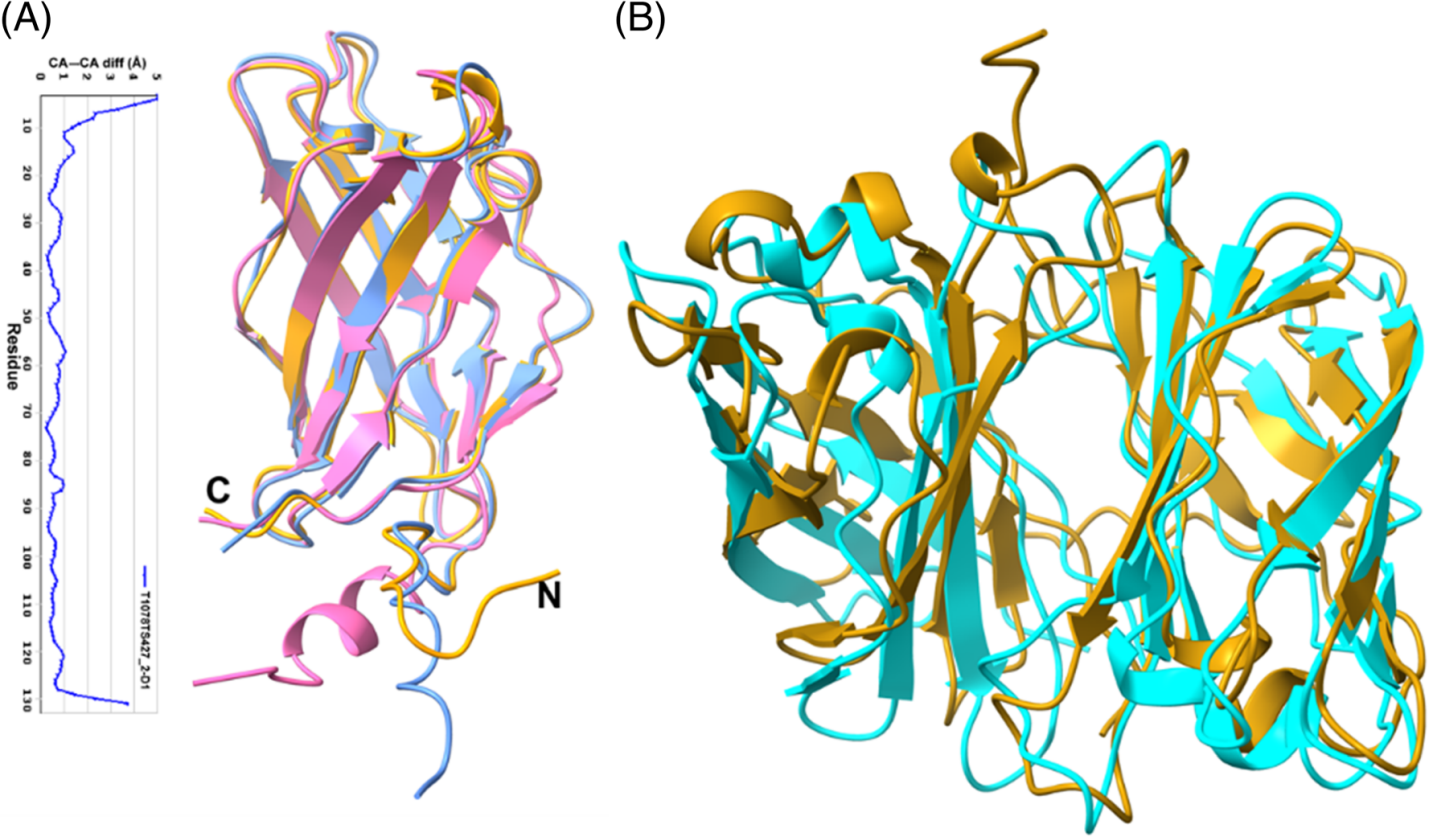
(A) Superposition of the Tsp1 monomer crystal structure (golden yellow) with top two CASP14 models T1078TS427_2‐D1 (blue) and T1078TS314_1‐D1 (pink). Also, given here is the plot of CA‐CA distance (in Å) between crystal structure and CASP14 model T1078TS427_2‐D1. (B) Comparison of dimeric structure of Tsp1 (golden yellow) with the best predicted multimeric model (T1078TS177_3o, cyan)

The results of CASP14 experiments are very interesting. To our surprise, the top ranked model by AlphaFold2 (Gr id 427, model T1078TS427_1‐D1) correctly reproduced the structure of the protein with RMSD of 0.96 Å and GDT‐TS of 95.93 for CA atoms (Figure 11A). Even the extended N‐ and C‐terminal regions with irregular secondary structure were predicted accurately, with more than 96% residues correctly aligned with the experimental structure. The accuracy in side chain rotamer predictions was also very good with RMS_all of 1.7 calculated on all atoms. Though the di‐sulfide bonded cysteines are placed juxtapose to each other in the predicted structure but the di‐sulfide linkages have not been predicted. Other top ranked models from FEIG‐R1 (GR# 314), FEIG‐R2 (GR# 480), FEIG‐S (GR# 013s), and Seder2020hard (GR# 428) groups also predicted the protein fold correctly with GDT score more than 80 (Figure 11A). Tsp1 forms dimer and the dimeric interface was also predicted with significant accuracy by Zou group (Gr id 177, model T1078TS177_3o) with the RMSD of 2.5 Å between target and model inter‐chain interface residues (Figure 11B). The contact agreement score, QS (best) score, of the prediction was 0.78. The interfaces in the models predicted by other groups had large RMSD values, and the inter subunit contact residues are not aligned with the experimental structure.

### 2.10 Histidine zipper coiled coils (CASP: T1083, T1084, T1087; PDB: N/A). Provided by Marcus D. Hartmann and Andrei N. Lupas

α‐Helical coiled coils are among the most ubiquitous and best‐understood protein folds.^105^ They are bundles of at least two α‐helices with a specific and repetitive packing and architecture, in which the interface between helices is typically dominated by hydrophobic residues, especially leucine.^106^ Based on initial speculations that the hydrophobic residues of the individual helices would interdigitate like the teeth of a zipper, short coiled coils are also often termed leucine zippers,^107^ although the eponymous hypothesis shattered when the first crystal structures showed that the hydrophobic residues are not interdigitating at the interface, rather being arranged like the rungs of a ladder. In recent years, however, we have come across a family of coiled‐coil proteins that essentially resembles the initially hypothesized zipper architecture, although with a decisive difference. This family is especially rich in histidines, which are found in a repetitive arrangement and it is these histidines that interdigitate like the teeth of a zipper between two antiparallel helices of a monomeric α‐helical hairpin.^108^ As seasoned coiled‐coil researchers, we set out to further characterize and delineate this unexpected new coiled coil flavor.

In sequence searches we identified a wide range of such histidine zippers. All of them appeared to form hairpins of different types, which we confirmed with the determination of several crystal structures. Interestingly, many of them turned out to be homo‐oligomers, in which a histidine‐zipper interface can be found either within the monomers (intra‐chain), between the monomers (inter‐chain), or both. We expected these to be possibly challenging targets for structure prediction and proposed three representatives for CASP14, one from *M. tundripaludum* (Tuna, T1087, Uniprot: G3J1N2), one from *N. oceani* (Nitro, T1083, Uniprot: Q3JAX3), and one from *M. silvanus* (Meio [homophonic to “mayo”], T1084, Uniprot: D7BIZ4). While all of them form antiparallel homo‐dimers, their histidine zipper interfaces are found in different forms and places. In Tuna, only the intra‐chain interface is a perfect histidine zipper. Nitro has an interface architecture similar to Tuna, but has most of the histidines replaced by tyrosines. Meio finally sports perfect intra‐ and inter‐chain histidine zippers. Currently, we can only speculate about the functional role of these proteins, and hypothesize that they might function as scavengers of metal ions.

To our surprise, most groups and servers did a very good job at predicting this new variant of the coiled‐coil fold. It is likely that several predictors have benefitted from the structure of the first representative that we had published for this fold previously, from the fungus *Serendipita indica* (PDB: 5LOS).^105^ This instance has 23% sequence identity to Tuna, 15% to Nitro and 19% to Meio. However, it was not identified as a template by the CASP prediction center for either of the three targets, and also sequence searches with HHpred^109^ using the standard settings of the MPI Bioinformatics Toolkit (as of March 2021)^110^ do not identify it as a template for every target. Nevertheless, each of the three targets was predicted with a GDT‐TS > 50 by more than 100 groups and with a GDT‐TS > 80 by more than 30 groups. Overall, the very best prediction was for Tuna, with a GDT‐TS of 96.8 and a GDT‐HA of 92.2, provided by AlphaFold2. For Meio, the best GDT‐TS of 93.0 was achieved by the server BAKER‐ROSETTASERVER, and the best GDT‐HA of 85.9 by BAKER‐experimental. For nitro, four predictors achieved the same best GDT‐TS of 87.8 (ropius0, ropius0QA, CAPRI‐Shen, and the server RaptorX), while the best GDT‐HA of 75.0 was achieved by AlphaFold2.

**FIGURE 12:**
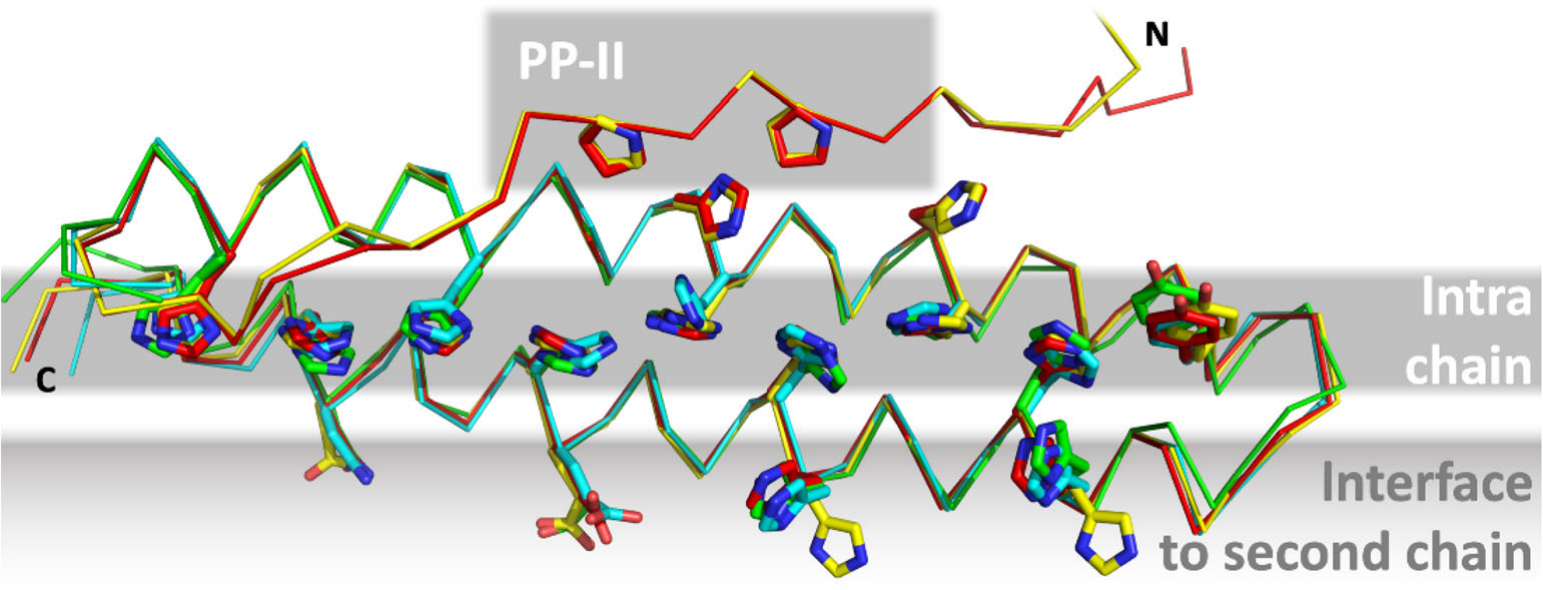
Single chain of the Tuna (T1087) crystal structure (yellow) superimposed with exemplary predictions (red: AlphaFold2; cyan: FEIG‐S, green: BAKER‐ROBETTA). Tuna forms two coiled‐coil interfaces, one intra‐chain and one for homo‐dimerization with another chain. Although only the monomer was a prediction target in CASP 14, several residues of the inter‐chain interface were predicted in the correct rotamer by the top‐ranking groups and servers. The N‐terminal extension containing a polyproline‐II helix is only shown for AlphaFold2, which predicted it most precisely in conformation and position

The most important feature of all three targets, the correct orientation of the histidines to form the zipping interactions was generally predicted very well in the top predictions, even in those from the best servers. According to the CASP14 evaluation formula, which we describe in a separate article in this special issue,^111^ the best server predictions for Tuna were the ones by FEIG‐S and BAKER‐ROBETTA—they are depicted together with the AlphaFold2 prediction and the crystal structure in Figure 12. One detail, however, was only predicted correctly by AlphaFold2: actually unrelated to the histidine zipper fold, the N‐terminal extension of Tuna, seen just in a few homologs from *Methylobacter* species, forms a polyproline‐II helix that buttresses the N‐terminal α‐helix, similar to an interaction engineered by Woolfson and colleagues into a stable miniprotein, PDB: 5LO3.^112^

### 2.11 Duck hepatitis B core protein (CASP: T1099, PDB: 6YGH). Provided by Cihan Makbul and Bettina Böttcher

Worldwide more than 250 million people are chronic carriers of hepatitis B virus (HBV) and have an increased risk for developing liver cancer or liver cirrhosis. Despite vaccination programmes, about 900 000 people die each year from HBV infection and related complications.

HBV is an enveloped virus that belongs to the family of Hepadnaviridae. This ancient family evolved more than 400 million years ago and is found in nearly all vertebrates.^113^ Hepadnaviridae form enveloped viruses with a lipidic envelope that is densely packed with surface proteins. This envelope surrounds an icosahedral capsid of 240 copies of hepatitis B core protein (HBc) and contains a viral polymerase together with the viral genome.

For many years, duck hepatitis B virus (DHBV) has been used as a model system for studying HBV infection. However, DHBV and human HBV (huHBV) belong to different linages of the Hepadnaviridae, namely, avihepadnavirusses and orthohepadnavirusses. These lineages differ in size and sequences of their viral proteins. In particular, the capsid forming HBc is much larger in avihepadnavirusses than in orthohepadnavirusses.

**FIGURE 13:**
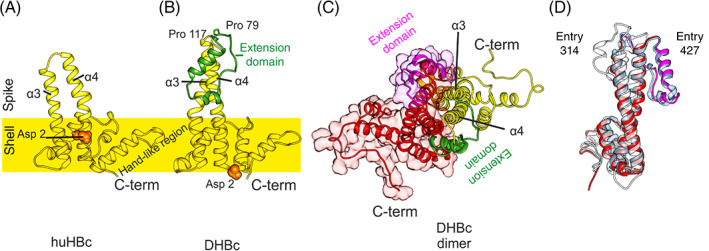
Structures of DHBc PDB: 6YGH^114^ and huHBc PDB: 6HTX.^115^ (A) View of the huHBc monomer perpendicular to the dimer axis. The approximate position of the capsid shell is indicated to mark the protruding part of the spikes. Each monomer contributes the helices α3 and α4 to the spike and dimer interface. The C‐terminus and the hand‐like region mark the inter‐dimer contact sites. The N‐terminus (Asp2, orange spheres) is part of the intra dimer interface in huHBc (A) but not in DHBc (B). (B) View of the DHBc monomer perpendicular to the dimer axis. The extension domain is delineated by Pro79 and Pro119 and is shown in green. (C) View of the DHBc dimer perpendicular to the dimer axis. The other monomer is shown in red/magenta. The two extension domains (magenta and green) are part of the dimer interface and broaden the core spike. (D) View onto the dimer interface of one monomer (red/magenta in [C]), with the superimposition of the two best scoring predicted models (entry 427, group AlphaFold2 in light blue; entry 314, group FEIG‐R1 in white)

In both lineages, HBc consists of a predominantly α‐helical, N‐terminal assembly domain (Figure 13) that forms the capsid, and an unstructured arginine‐rich C‐terminal domain (CTD) that projects into the capsid interior and fine‐tunes the charge balance with the genome. Only the ordered assembly domain of HBc has been amenable to structure determination, and huHBc has been studied for decades .^115^, ^116^, ^117^ The assembly domain of huHBc forms hammer‐shaped dimers that assemble into capsids with protruding spikes,^118^ and these spikes contact the envelope in viruses and virus‐like particles.^119^, ^120^

Each monomer contributes two long helices (α3 and α4), connected by a short loop, to the intra‐dimer interface of the spikes (Figure 13A). The inter‐dimer contacts are mediated by a hand‐like region that follows the helical hairpin in the spike and precedes the CTD. The sequences of inter‐dimer contacts are conserved among Hepadnaviridae, which is not the case for the inner dimer contacts, or the protruding part of the spikes.

In contrast to huHBc, DHBc is much larger with an extension domain of approximately 40 residues that maps to the loop region of the spikes. To understand the structural importance of this extension domain, we determined the structure of DHBc in capsids by electron cryo microscopy.^114^ As in huHBc, the core of the spike is formed by a four helical bundle with two helices from each monomer (Figure 13). These helices are longer than in huHBC with a different twist and tilt leading to changes of their relative positions at the tips of the spikes.

The extension domain contains a long and a short helix and replaces the short connecting loop between α3 and α4 in huHBc. The domain folds at the side of the spikes, where it enlarges the intra‐dimer interface. This is further enhanced by a salt bridge between R124 in the core spike and E109 in the extension domain, which is essential for immobilizing the extension domain at the side of the spikes and increasing the capsid stability.^114^

The extension domain contains eight prolines, two of which are close to its C‐ and N‐termini, separating the extension domain from the core‐spike (Figure 13B). Although the extension domain contributes to the dimer interface, it is dispensable for capsid formation and folds slowly over weeks in the *E. coli*‐expressed protein. This slow folding depends on the cis‐trans isomerization of some of the eight prolines and can be enzymatically accelerated with a peptidyl‐prolyl cis‐trans isomerase. In the folded state, the close proximity between C‐ and N‐termini of the extension domain generates a cleft close to the tip of the spike that resembles the binding motif between the two short loops in the huHBc spike. Thus, the extension domains provide two potential‐binding sites per spike, where huHBc has only one.

Many of the CASP14 models reproduced the main features of the DHBc‐monomer correctly: they identified the longer spike helices, the fold of the extension domain with a longer and a shorter helix, as well as the position of the extension domain at the side of the spikes. However, the twist and tilt of the helices in the upper half of the core spike and the relative position of the extension domain in respect to the core spike were not correctly predicted. One exception was the model T1099TS427_1‐D1 from AlphaFold2. Here, the tilt and the twist in the core spike were modeled correctly (QCS = 98) and the extension domain was properly placed in respect to the potential dimer interface.

Some of the CASP14 predictors provided models of the DHBc dimer. The models correctly reproduced the dimer interface (QS = 0.45) in the core spike but missed the correct twist of the helices around each other. Therefore, the extension domain was misplaced and did not contribute to the dimer interface. Many predicted models also diverged from the experimental model in the position of the N‐terminal 10–15 residues. While the helical fold in this region was correctly predicted, it was wrongly placed at the dimer interface at the base of the spike. This orientation is similar to what is observed in huHBc, with the N‐terminus embracing the spike and packing against the opposite monomer at the base of the spike. However, in the experimental structure of DHBc the N‐terminal helix is not a part of this dimer interface and points toward the capsid interior (Figure 13).

In conclusion, many predictions recapitulated key‐features of the fold of DHBc but failed to predict changes in the oligomerization interfaces that deviated from huHBc.

### 2.12 Cancer biology and the ASCC1 alkylation response protein structure by Naga Babu Chinnam, John A. Tainer, and Susan E. Tsutakawa (CASP: T1101, PDB: N/A)

Originating from medical studies on the warfare use of mustard gas in World War I, alkylation chemotherapy is among the most widely used forms of systemic therapy for cancer today.^121^, ^122^ Its damage to DNA and subsequent disruption of replication in cancer cells was thought to the primary reason for its efficacy against cancer. Structure‐based design of alkylation repair inhibitors promised to reduce resistance to alkylation chemotherapy and started over 20 years ago with the work on alkyl‐guanine transferase (AGT or MGMT).^123^, ^124^, ^125^ Yet, applications of targeting alkylation repair have lagged behind other ways to target the DNA damage response, where structural and mechanistic knowledge of proteins that repair DNA damage spanning from base damage to single and double‐strand breaks have provided insights into cancer etiology: Prognosis, sensitivity, and resistance.^126^, ^127^, ^128^, ^129^, ^130^, ^131^, ^132^, ^133^, ^134^, ^135^, ^136^, ^137^, ^138^

Despite extensive use of alkylating agents in cancer medicine, we still do not adequately understand what alkylation chemotherapy is doing. Studies showed that prostate cancers overexpress ALKBH3 and noted that overexpression is related to the metastatic cancers with poor prognosis.^139^, ^140^, ^141^, ^142^, ^143^, ^144^ That downregulation of ALKBH3 sensitized cancer cells but not normal cells to alkylating agents suggested that this dependency on ALKBH3 is cancer‐specific.^139^ As ALKBH3 is a single strand‐specific dealkylating enzyme for both DNA and RNA, these results suggested that RNA damage contributed to the chemotherapeutic effectiveness of alkylating chemotherapies. We know from other DNA repair systems that understanding protein partners is critical to a structural and mechanistic knowledge of the DNA damage response. For ALKBH3, key partners come the Activation Signal Cointegrator Complex (ASCC, also known as ASC‐1), composed of three subunits. ASCC3 has two DEAD box helicase domains.^139^ ASCC2 has a CUE (coupling of ubiquitin conjugation to ER degradation) domain.^145^ ASCC1 has two domains, an RNA‐binding KH domain and a domain orthologs to AKAP18, a phosphoesterase domain which binds AMP.^146^ To better understand what the ASCC ALKBH3 partners are doing in response to alkylating agents, we initiated structural analysis. We crystallized ASCC1 from *A. pompejana*, a hyperthermophilic animal whose proteins typically show high sequence similarity to human proteins, are amenable to crystallization, and diffract to high resolution, as evidenced by superoxide dismutase sequence and structures.^147^

**FIGURE 14:**
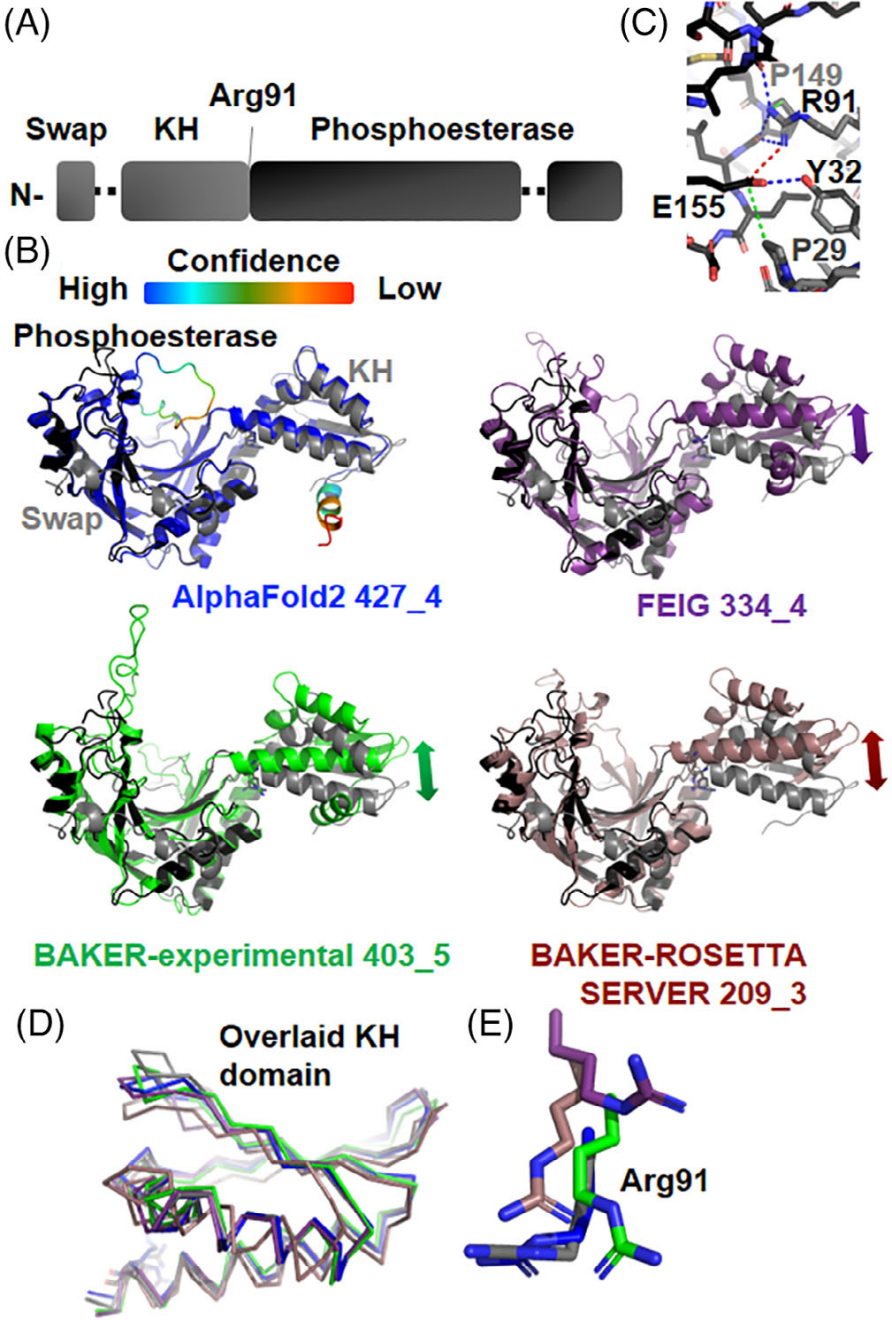
Target 1101 ASCC1. Numberings are based on prediction models with a difference from the protein by −40. (A) Domain schematic shows sequence position of domain swap, KH domain, and phosphoesterase domain. (B) ASCC1 shows KH and phospoesterase domain positioned side‐by‐side. Prediction models (color) were overlaid pairwise on the phosphoesterase domain of the ASCC1 experimental crystal structure (colored as in [A]). The AlphaFold2 model was colored based on the confidence level of the prediction (given in color bar). Arrows indicate significant deviation in domain position. (C) A zoomed view of the interface between the KH and phosphoesterase domain. Red, blue, and green indicate cross‐domain salt bridge, hydrogen bonding, and van der Waals, respectively. (D) Overlay of KH domains with crystal structure. Color is same as in (B). (D) A zoomed view of Arg91, positioned at the center of the KH‐phosphoesterase domain interface. Three of the four models positioned it accurately. Color is same as in (B). (E) A zoomed view of Arg91, positioned at the center of the KH‐phosphoesterase domain interface. In three of the four models, it was positioned accurately. Color is same as in (B)

The X‐ray crystal structure of *A. pompejana* ASCC1 was to 1.4 Å with one molecule in the asymmetric unit (Figure 14A,B). Its domains had mixed alpha/beta folds. The RNA‐binding K‐homology (KH) domain has 1–2 Å RMSD to other KH domains known for binding 4 nt RNA with sequence specificity that varies according to the protein. The phosphoesterase domain has a structural similarity of 1–3 Å RMSD to phosphoesterase and RNA‐ligase domains. This phosphoesterase superfamily has two invariably conserved HXT motifs, whose function is mostly unknown. The stacking of one HXT motif against the cyclic mononucleotide ligands in the AKAP18 structures suggests a role in substrate recognition, although structural mechanisms and activity remain enigmatic for this family. The two domains are oriented along one axis relative to each other. In the crystal structure, the partially helical N‐terminus does a domain swap and packs along the side of the central beta sheet of the phosphoesterase domain.

Although the two domains of ASCC1 have structural orthologs, we thought that the structure would be interesting for CASP to see if prediction algorithms would predict the domain‐domain orientation and an N‐terminal domain swap. There are no orthologs with this combination of domains. The interface between the two domains is not extensive (Figure 14C). At the center of the interface, there is a conserved arginine 91 on the KH domain, making a salt bridge to a glutamate 155, H‐bonding to two main chain contacts, and packing against a proline 149 on the phosphoesterase domain. The phosphoesterase domain glutamate 155 has also hydrogen bonding to a tyrosine 32 and packing against a proline 29 on the KH domain. Finally, the aliphatic chain of a KH domain arginine 91 packs against that of an asparagine. With respect to the domain swap, the interface is more extensive and the N‐terminal domain contains one region with highly conserved residues. In this region, a phosphoesterase domain tyrosine hydrogen bonds to a backbone carbonyl and packs against a phenylalanine and the main chain in the N‐terminal region. This N‐terminal region phenylalanine plus a nearby leucine are also packing against a leucine, arginine, and main chain in the phosphoesterase domain. Two conserved arginines in the N‐terminal region pack against main chain in the phosphoesterase domain.

Importantly, the top scoring models from AlphaFold2 (GDT‐TS = 88/IDDT = 0.86), BAKER‐experimental (GDT‐TS = 68/IDDT = 0.71), and FEIG (GDT‐TS = 63/IDDT = 0.70) groups as well as one from the top scoring server, BAKER‐ROSETTASERVER (GDT‐TS = 61/IDDT = 0.70), predicted the relative orientation of the two domains surprisingly well with at most a 20° rotation offset (Figure 14). AlphaFold2 distinctly predicted the orientation of the two domains in all five submitted models, while the predictions of other three groups were closer to each other than to the crystal structure. At one end of the KH domain beta sheet, the AlphaFold2 model diverged from the crystal structure by as much as 5 Å, when the phosphoesterase domain was overlaid, or 3.5 Å when the KH domain was overlaid (Figure 14B,D). These models had a remarkable CA‐RMSD of 3.1, 3.6., 5.1, 5.0, and 5.0 Å over all residues, respectively. Based on our examination of the interface, the Arg91 position (numbered based on the prediction model numbering) is critical. Arg91 from three of four models overlaid onto the corresponding residue in the crystal structure. The FEIG model had Arg91 shifted away by 5 Å. In the crystal, Arg91 was in two alternative positions, and the side chain AlphaFold2 Arg91 almost exactly overlaid onto one of the Arg91 positions (Figure 14E).

As expected, the prediction models did not converge on a similar position for regions that could not be modeled in the electron density. The AlphaFold2 team provided residue‐by‐residue confidence scores, and low confidence regions matched the two loop regions unable to be modeled in the experimental electron density. None of the models predicted the crystallographic position of the N‐terminus, and the AlphaFold2 team scored this region as low confidence. With these results, we reconsidered the crystallographic model. Since the N‐terminal region is disconnected from the KH domain, we cannot exclude the possibility that the N‐terminal region observed is a part of another molecule in the crystal lattice. Either the domain swaps could not be predicted correctly, or the N‐terminal region is actually a crystallographic artifact. Thus, the computational models may prove an accurate guide for further studies.

While previous SAXS studies that directly measure flexibility^148^ suggested that, in general, X‐ray structures were too rigid,^149^ computational predictions were uncovering the greater flexibility of the solution structures.^150^, ^151^ Indeed, several repair proteins were shown to be functionally flexible,^129^, ^152^ and our X‐ray structure revealed a simple loop connecting the two domains, consistent with substantial flexibility between the two domains.

Yet, the clear consensus of the highest ranked prediction models on the relative orientation of the two domains suggests to us that the ASCC1 domains are not flexible relative to each other but are rigidly encoded in the sequence. Perhaps, ASCC1 activity is strictly controlled and that this rigidity plays a role in the regulatory mechanism. So the prediction models and their interesting implications will be tested by SAXS and mutational analyses, which ultimately need to be integrated with testing in and structural imaging in cells that can provide the most relevant environment,^153^, ^154^ Furthermore, emerging cancer biology data are showing that it is important to understand the structure of the nucleic acid as well as of the damage response proteins.^155^ So, the potential structural rigidity of ASCC1 suggests its activity may favor specific RNA structures or serve to sculpt RNA for cleavage. Overall, the computational predictions were accurate, useful, and can help guide ongoing and future experiments.

## 3 CONCLUSIONS

This article describes the structural and functional aspects of the selected CASP14 targets. The authors of the structures highlighted the most interesting target features that were reproduced in the models, and also discussed the drawbacks of the predictions. The overall ability to predict three‐dimensional structures of proteins has improved remarkably, and many difficult targets were modeled with impressive accuracy.

When modeling monomeric targets, AlphaFold2 systematically outperformed other methods, followed up by runners‐up in predicting some targets, and the authors suggested that the top models could be used to confidently infer functional sites of the protein. For example, for target T1057, top two predictions would allow for correct assignment of active site catalytic residues and environment.

There is, however, room for improvement when it comes to modeling loops. It also remains challenging to accurately model multimeric protein complexes. In some cases, the limiting factor could be the lack of the adequate structure of the individual components (e.g., targets H1036 and H1065). In other cases, predictions of the individual components were highly accurate, yet the methods failed to reproduce the relative orientations observed in their oligomeric states. Examples include incorrect oligomerization interface of the DHBc spike (T1099), and large deviations of the ring assembly for the phage T5 tail tip complex, where no model was able to reproduce inter‐ring distances and diameter (H1060 and T1061). We also observed that the conformations of the models for several targets, for example, T1054, T1068, and T1101, differed from the experimentally determined structures. As the authors pointed out, these conformations may represent alternative biologically relevant states, and could be helpful for better understanding of the structural dynamics of the targets.

The outcomes of this critical assessment have paved the way for increasing the synergies between computational and experimental approaches to protein structure determination. As described in another article of this issue, several of the CASP14 targets were solved with the aid of the models, or the models allowed to improve structure accuracy.^7^ The synergies could be particularly helpful for capturing conformations that may eluded experimental structure determination, particularly in membrane proteins,^156^ or as a strategy for attempting molecular replacement phasing that has already been shown to be beneficial.^157^

In conclusion, we have shown that for the targets described here, the most critical structural features were accurately reproduced by the models. The experimentalists now foresee the models guiding further studies of biologically‐relevant properties of proteins, including spatial orientations of structural elements and their dynamics. The performance of computational methods has increased, so has the confidence in the scientific value of the results they produce.

## AUTHOR CONTRIBUTIONS

*Concept, abstract, introduction, editing, and coordination*: Leila T. Alexander, Rosalba Lepore, Andriy Kryshtafovych, Krzysztof Fidelis, John Moult, Maya Topf, and Torsten Schwede. Target‐specific sections—by authors provided in the sections' titles.

### PEER REVIEW

The peer review history for this article is available at https://publons.com/publon/10.1002/prot.26247.

## Supporting information


**Table S1** CASP14 target providers
**Table S2**. Best templates for the described targets

## Data Availability

Data sharing not applicable to this article as no datasets were generated or analysed during the current study.
