# Supplementary material for: Target highlights in CASP14: Analysis of models by structure providers
Source: Proteins. 2021 Oct 10;89(12):1647–72. doi: 10.1002/prot.26247 (PMC8616854; doi:10.1002/prot.26247)
Supplement: Supplementary file 1 — Table S1 CASP14 target providers Table S2. Best templates for the described targets [file PROT-89-1647-s001.pdf]

## SUPPLEMENTARY INFORMATION

# Target highlights in CASP14: analysis of models by structure providers

### Correspondence

Leila Tamara Alexander, email: leila.alexander@sib.swiss

**TABLE S1.** CASP14 target providers

| # | Contributor | Country | Institution | Target |
|---|---|---|---|---|
| 1 | Athanassios Adamopoulos | Netherlands | Cancer Institute | T1068 |
| 2 | Markus Alahuhta | USA | NREL | T1057 |
| 3 | Ann M. Arvin | USA | Stanford U | H1036 and T1036 |
| 4 | Yannick J. Bomble | USA | NREL | T1057 |
| 5 | Bettina Böttcher | Germany | Julius-Maximilians U Würzburg | T1099 |
| 6 | Cécile Breyton | France | IBS, Grenoble | H1060 and T1061 |
| 7 | Naga Babu Chinnam | USA | U of Texas | T1101 |
| 8 | Valerio Chiarini | Finland | U of Helsinki | T1034 |
| 9 | Wah Chiu | USA | Stanford U | H1036 and T1036 |
| 10 | Rhys Grinter | Australia | Monash U | T1054 |
| 11 | Gagan D. Gupta | India | BARC | T1078 |
| 12 | Marcus D. Hartmann | Germany | Max Planck, Tübingen | T1083, T1084, T1087 |
| 13 | Christopher S. Hayes | USA | U California, Santa Barbara | H1065, T1065s2, T1065s1 |
| 14 | Tatjana Heidebrecht | Netherlands | Cancer Institute | T1068 |
| 15 | Andrea Ilari | Italy | CNR | T1034 |
| 16 | Andrzej Joachimiak | USA | U of Chicago | H1065, T1065s2, T1065s1 |
| 17 | Youngchang Kim | USA | U of Chicago | H1065, T1065s2, T1065s1 |
| 18 | Romain Linares | France | IBS, Grenoble | H1060 and T1061 |
| 19 | Andrew L. Lovering | UK | U of Birmingham | T1074 |
| 20 | Vladimir V. Lunin | USA | NREL | T1057 |
| 21 | Andrei N. Lupas | Germany | Max Planck, Tübingen | T1083, T1084, T1087 |
| 22 | Cihan Makbul | Germany | Julius-Maximilians U Würzburg | T1099 |
| 23 | Karolina Michalska | USA | U of Chicago | H1065, T1065s2, T1065s1 |
| 24 | Prasun K. Mukherjee | India | BARC | T1078 |
| 25 | William (Sam) Nutt | USA | U of Chicago | H1065, T1065s2, T1065s1 |
| 26 | Stefan L. Oliver | USA | Stanford U | H1036 and T1036 |
| 27 | Anastassis Perrakis | Netherlands | Cancer Institute | T1068 |
| 28 | Lucy Stols | USA | U of Chicago | H1065, T1065s2, T1065s1 |
| 29 | John A. Tainer | USA | U of Texas | T1101 |
| 30 | Susan E. Tsutakawa | USA | LBL | T1101 |
| 31 | Mauricio Valdivia-Delgado | UK | U of Birmingham | T1074 |

**TABLE S2.** Best templates for the described targets

| Target | Best template | Coverage, % | Sequence identity, % |
|---|---|---|---|
| T1034 | 6qaz_A | 79.49 | 26.61 |
| T1036s1 | 6esc_A | 97.77 | 61.58 |
| T1060s2 | 5ngj_A | 74.41 | 5.88 |
| T1060s3 | 6v8i_BK | 72.36 | 17.98 |
| T1061-D1 | 1pdj_D | 54.09 | 8.76 |
| T1061-D2 | 2yc2_B | 25.09 | 10.29 |
| T1061-D3 | 6bx4_D | 76.7 | 17.72 |
| T1065s1 | 3hi2_D | 54.62 | 9.23 |
| T1065s2 | 5l0c_D | 67.35 | 16.67 |
| T1054 | 4qo6_A | 51.75 | 20.27 |
| T1057 | 2dpm_A | 86.4 | 30.56 |
| T1068 | 2xse_A | 59.22 | 23.58 |
| T1074 | 3wjc_A | 67.42 | 8.99 |
| T1078 | 3v0r_A | 71.32 | 9.78 |
| T1083 | 6grj_D | 91.3 | 11.9 |
| T1084 | 1afr_E | 95.77 | 11.76 |
| T1087 | 5vju_A | 92.47 | 17.44 |
| T1099 | 5gmz_B | 56.91 | 26.17 |
| T1101-D1 | 2ann_A | 84.34 | 27.14 |
| T1101-D2 | 1vdx_A | 74.77 | 17.5 |
